# Atomic view of the histidine environment stabilizing higher-pH conformations of pH-dependent proteins

**DOI:** 10.1038/ncomms8771

**Published:** 2015-07-20

**Authors:** Céline Valéry, Stéphanie Deville-Foillard, Christelle Lefebvre, Nuria Taberner, Pierre Legrand, Florian Meneau, Cristelle Meriadec, Camille Delvaux, Thomas Bizien, Emmanouil Kasotakis, Carmen Lopez-Iglesias, Andrew Gall, Stéphane Bressanelli, Marie-Hélène Le Du, Maïté Paternostre, Franck Artzner

**Affiliations:** 1 Biomolecular Interaction Centre, School of Biological Sciences, University of Canterbury, 8140 Christchurch, New zealand; 2 Ipsen, 5 Avenue du Canada, 91940 Les Ulis, France; 3 CEA, Institute of Biology and Technologies of Saclay, 91191 CEA-Saclay, France; 4 Institute for Integrative Biology of the Cell (I2BC), CEA, CNRS, Université Paris-Sud, 91191 CEA-Saclay, Gif sur Yvette, France; 5 CNRS, UMR 6251, Institut de Physique de Rennes, 263 av. Général Leclerc, Université Rennes I, 35042 Rennes Cedex, France; 6 Synchrotron SOLEIL, 91190 Gif sur Yvette, France; 7 Cryo-Electron Microscopy Unit. Scientific and Tecnological Centers of the University of Barcelona, E-08028 Barcelona, Spain

## Abstract

External stimuli are powerful tools that naturally control protein assemblies and functions. For example, during viral entry and exit changes in pH are known to trigger large protein conformational changes. However, the molecular features stabilizing the higher pH structures remain unclear. Here we elucidate the conformational change of a self-assembling peptide that forms either small or large nanotubes dependent on the pH. The sub-angstrom high-pH peptide structure reveals a globular conformation stabilized through a strong histidine-serine H-bond and a tight histidine-aromatic packing. Lowering the pH induces histidine protonation, disrupts these interactions and triggers a large change to an extended β-sheet-based conformation. Re-visiting available structures of proteins with pH-dependent conformations reveals both histidine-containing aromatic pockets and histidine-serine proximity as key motifs in higher pH structures. The mechanism discovered in this study may thus be generally used by pH-dependent proteins and opens new prospects in the field of nanomaterials.

## Abstract (web summary)

In biological systems, large pH-induced conformational changes can be observed in certain proteins, a phenomenon poorly understood at the molecular level. Here the authors describe a peptide with the ability to self-organize into either small or large nanotubes in a pH-dependent manner and detail the mechanism driving the transition.

## Graphical abstract

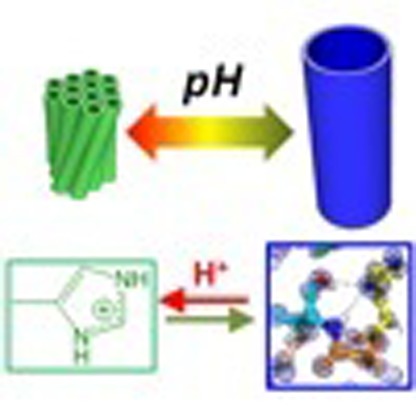

Proteins commonly undergo changes in their three-dimensional (3D) structures to perform their functions. Biologically induced conformational changes can involve motions of groups of atoms or even whole domains of proteins. Changes in protein shape can be triggered by charges, such as calcium and phosphate^1^, or protonation in pH-dependent activities^2^. The detailed comprehension of the underlying molecular mechanisms is an important contribution to the general understanding of protein function. For instance, a wide range of viruses take advantage of the low pH found in host cell endosomes to sense their cellular location and become activated at the right time and place, for example, to mediate viral and host membrane fusion. The viral proteins responsible for this process have been widely reported to commonly undergo large-scale pH-triggered conformational and oligomerization changes during the membrane fusion process. This is the case for varied pathogens such as influenza virus, vesicular stomatitis virus^3^,^4^, tick-borne encephalitis virus^5^,^6^ and dengue^7^,^8^ virus. Histidine protonation is suspected to be involved in the molecular switch that induces the protein pH-dependent motions. However, experimental demonstrations of single histidines as biomolecular switches have been few, indicating that the precise molecular mechanisms of the protein conformational changes are not well understood, particularly the molecular determinants that stabilize the high-pH protein conformation and trigger its destabilization^9^,^10^.

In addition, the perfect size monodispersity and the responsiveness of these biological self assemblies correspond to the major challenges for future advanced nanomaterials, that is, (i) the predictable control of the size and morphology of the self-assembled architectures by the chemical structure of the building blocks and (ii) the design of versatile systems that can be triggered by external stimuli. The monodispersity of the final organization and the accuracy of the bio-inspired self-assembly mechanisms have recently attained quasi-perfection with various biological building blocks, such as nucleic acids and amino acids. Any two-dimensional (2D) or 3D nano-object can be designed with DNA origamis^11^,^12^,^13^, while DNA-responsive architectures have been obtained, such as a nano-sized box that can be locked or opened in response to a short-single-stranded DNA^14^. Responsiveness is, however, limited by the DNA interaction and functionalization possibilities. In this respect, RNA or mixed RNA/DNA origami may be more promising thanks to RNA softness and aptamer recognitions^15^,^16^. The potential of amino acids for the construction of responsive nanomaterials is correspondingly huge and yet to be tapped. Proteins and peptides are made of building blocks and secondary structures exhibiting a huge variety of interactions and responsive functions. Indeed, proteins^17^,^18^ and peptides can be rationally modified to control the self-assembly shapes^19^,^20^,^21^,^22^,^23^,^24^,^25^,^26^ as well as to finely tune the diameter of nanotubes^22^,^23^,^24^.

In this letter, we report on a decapeptide that self-assembles into either 50 nm diameter nanotubes at high pH or 10.7 nm diameter nanotubes at low pH. The peptide conformation switches from a globular one at pH>7.5 to an extended one at pH<6.5. The high-pH crystal structure obtained at 0.85 Å resolution reveals a histidine-serine H-bond and histidine-aromatic interactions, whereas the low-pH molecular structure demonstrates these key interactions have disappeared, likely in favour of cation-π proximities. Interestingly, reanalysing protein structures with pH-dependent functions reveals that these specific interaction networks are present in viral, bacterial and human proteins. This molecular switch is demonstrated to have a high potential for the bottom-up design of pH-responsive nanomaterials.

## Results

### Conformation transition

**Figure 1:**
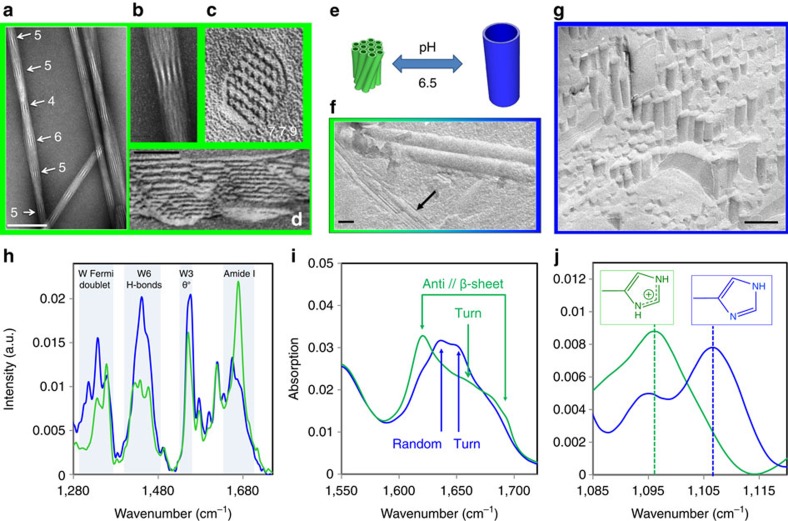
pH-dependent peptide self-assemblies. (**a**–**d**) Electron micrographs of triptorelin self-assemblies formed at low pH (pure water, pH 6), (**a**,**b**) negative staining and (**c**,**d**) freeze fracture. (**a**) Bundles of small nanotubes. The white arrows underline on one example the moire patterns on a nanotube bundle that reflect the regular twisting of the nanotubes within the bundles. Near the arrows are written the number of aligned nanotubes that are visible, that is, 5, 5, 4, 6, 5 in this case. (**b**) Zoom on the moire pattern showing five distinct aligned nanotubes. (**c**) Zoom on perpendicular fracture of a bundle containing 38 nanotubes. In the direction of the hexagonal packing 7, 7 and 9 alignments of nanotubes are visible. (**d**) Longitudinal fracture of a nanotube bundle. (**e**) Scheme underlying the differences of size and packing between a bundle of 12 small (low pH) and 1 large nanotube (high pH). (**f**) Electron micrographs of freeze-fracture replicas showing the coexistence between bundles of small (black arrow) and large nanotubes. See also Supplementary Fig. 10. (**g**) Electron micrographs of a freeze fracture replica of triptorelin large nanotubes formed at high pH (pH 8.5). (**h**) Comparison of the FT-Raman spectra of small (pH 6.5, green) and of large nanotubes (pH 8.5, blue). The spectra are the fingerprints of the peptide environment and interactions within the assemblies, especially for the environment of the W (Supplementary Fig. 2 and Supplementary Note 1). (**i**) ATR-FTIR Amide I spectra of small (pH 5.5, green) and large nanotubes (pH 8.5, blue). (**j**) ATR-FTIR spectra of small (pH 5.5, green) and large nanotubes (pH 8.5, blue). The absorption bands at 1,097 and 1,106 cm^−1^ are characteristic to (HisH_2_)^+^ (green) and (HisN_3_)H τ tautomer (blue), that is, protonation of N_3_, respectively. (electron micrograph scales: 50 nm).

Triptorelin is a decapeptide with uncharged N- and C-termini pE_1_–H_2_–W_3_–S_4_–Y_5_–(D)W_6_–L_7_–R_8_–P_9_–G_10_–NH_2_ that contains three aromatic side chains (Y_5_, W_3_ and W_6_), and three ionizable residues, R_8_ and Y_5_ (pKa>10) and a histidine, H_2_ (pKa=6.1 in water; Supplementary Fig. 1). In the following, the influence of pH is studied in the range 5–8, that is, with the R_8_ remaining positively charged for all the conditions here explored. The triptorelin peptide self-assembles in water into either twisted bundles of small nanotubes at low pH (pH<6.5, Fig. 1a–f) or large nanotubes at high pH (pH>7.5, Fig. 1g and Supplementary Fig. 3). Given that the peptide single histidine (H_2_) is the only residue with a pK_a_ in this range of pH, we explored its protonation state. As shown by the corresponding attenuated total reflectance Fourier-transformed infrared spectroscopy (ATR-FTIR) spectra (Fig. 1j), H_2_ is protonated as a positively charged imidazolium group in the low-pH assemblies (infrared absorption at 1,097 cm^−1^), whereas it is deprotonated in the high-pH assemblies, as a neutral and aromatic τ-tautomer^27^,^28^ mainly (infrared absorption at 1,106 cm^−1^, deprotonated histidine on the N_1_ nitrogen). From one structure to the other, the Fourier-transformed Raman spectroscopy (FT-Raman) evidences a major change in the environments of the aromatic residues and especially tryptophan (Fig. 1h, Supplementary Fig. 2a and Supplementary Note 1). The ATR-FTIR amide I spectra further reveal the presence of β-sheet hydrogen bond networks in the low-pH assemblies, whereas such networks are only weakly detected in the high-pH ones (Fig. 1i and Supplementary Fig. 2b and Supplementary Note 1). All together, these results show that the peptide conformation, the peptide molecular packing and the assembly morphology of the triptorelin self-assemblies depend on the protonation state of the peptide single histidine, H_2_ (Fig. 1e).

### High-pH nanotubes

**Figure 2:**
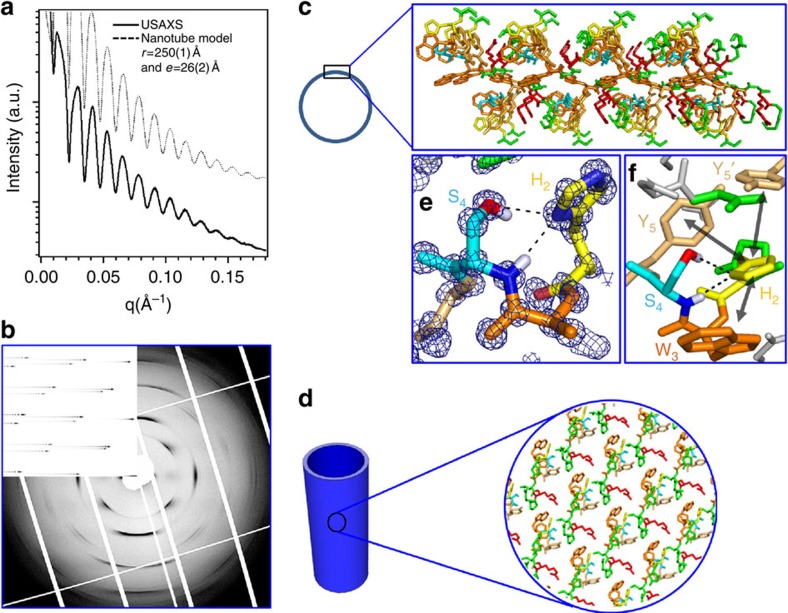
Atomic structure of the high-pH nanotubes. (**a**,**b**) Ultra small angle X-Ray scattering and aligned fibre diffraction pattern of the large nanotubes. (**c**,**d**) Atomic resolution of the peptide organization in the large nanotube wall: side (**c**) and top (**d**) views. Colour code: orange (W), light orange (Y), yellow (H), cyan (S) and red (R). The two layers of peptides that form the wall thickness are interacting via an extended aromatic stacking essentially formed by the W side chains. (**e**) Superposition of the electron density map and structure of the crystal zoomed on the H_2_–S_4_ H-bonds (2.1 Å between the N_1_ of the histidine and the proton of the serine OH group and 2.2 Å between N_1_ of the histidine and the proton of the amine of the serine backbone). (**f**) Interaction network around the H residue (yellow) within the peptide packing: H-bonds between H_2_ and S_4_ (black dashed lines) and aromatic cluster around H_2_ (H_2_-aromatic distance<4 Å) formed by W_3_ and two Y_5_: intra (Y_5_) and inter (Y′_5_) (black arrows).

At high pH (pH>7.5, H_2_ mainly as neutral aromatic τ tautomer), small-angle X-ray scattering (SAXS) reveals nanotubes with a monodisperse diameter (*Φ*) of 500 Å and a wall thickness (*e*) of 26 Å (Fig. 2a and Supplementary Fig. 3). In some cases, the 2D hexagonal packing of the nanotubes is observed with a lattice parameter (*a*_hex_) of 590 Å. The organization within the nanotube wall is crystalline as shown by an exceptionally well-aligned wide-angle X-ray scattering pattern (mosaicity≅5°) (Fig. 2b and Supplementary Fig. 4). This pattern can be interpreted in terms of a 2D-curved crystal. The position of diffuse scattering maxima can be indexed by a 2D monoclinic lattice *a*=13.7 Å, *b*=13.1 Å, *γ*=88.5°. The line shapes of the diffuse scattering can be simulated by Bessel functions *J*_*n*_ with *n*=88 × *h*+79 × *k* (Supplementary Fig. 4 and Supplementary Note 2) corresponding to the Fourier transform of a 2D lattice supported by a thin nanotube^25^.

### High-pH single crystal

To obtain insights into the nanotube structure at atomic level, the peptide was crystallized as used for amyloid structure determination^29^. The crystallization strategy was inspired by our previous investigations on the lanreotide nanotubes. We reported that specific steric hindrance modifications within the nanotube walls induced predictable variations of the lanreotide nanotube diameter^22^,^23^,^24^. When the lanreotide nanotubes reach a maximal diameter, the system tends to crystallize as flat lamellae packing, that is, with flat walls. Small triptorelin crystals were formed in the presence of phosphate anions that are larger than acetate. By optimizing the crystallization conditions large single crystals diffracting up to 0.85 Å resolution were obtained (Fig. 2e). The 2D-unit cell extracted from the large nanotube X-ray diffraction pattern (*a*=13.35 Å, *b*=12.93 Å and *γ*=88.5°) is fully compatible with the layer of the large crystals (*b/2*=13.75 Å, *c*=13.1 Å and *β*=90°) (Supplementary Figs 5 and 6, Supplementary Note 3, Supplementary Table 1). Moreover, the micro-Raman spectrum shows that the two W present in the peptide sequence are in two distinct environments and that there is no β-sheet network within the three types of assemblies, that is, large nanotubes, small and large crystals (Supplementary Note 4). This information allows us the use of the high-resolution atomic structure obtained by X-ray crystallography to decipher the structure of the wall of the large nanotube. Hence, the 25-Å-thick bilayer observed in the crystal structure (fully compatible with the 26 Å determined by SAXS) is essentially maintained by aromatic stacking, as shown by the side view of the bilayer (Fig. 2c) and in agreement with the low β-sheet content observed by ATR-FTIR (Fig. 1j). The histidine tautomer in the crystal and in the nanotubes is the τ tautomer. Remarkably, the histidine residue is part of a hydrophobic aromatic cluster (Fig. 2f and Supplementary Fig. 6) and its orientation is locked by two strong hydrogen bonds with the S_4_OH group and the backbone amine at distances (nitrogen to proton) of 2.1 and 2.21 Å, respectively (Fig. 2e). Finally, in agreement with both ATR-FTIR and Raman data, almost no direct intra- or inter-molecular hydrogen bonds stabilize the peptide conformation or the peptide packing except within the type II′ β-turn induced by the (D)W_6_.

### Low-pH nanotubes

**Figure 3:**
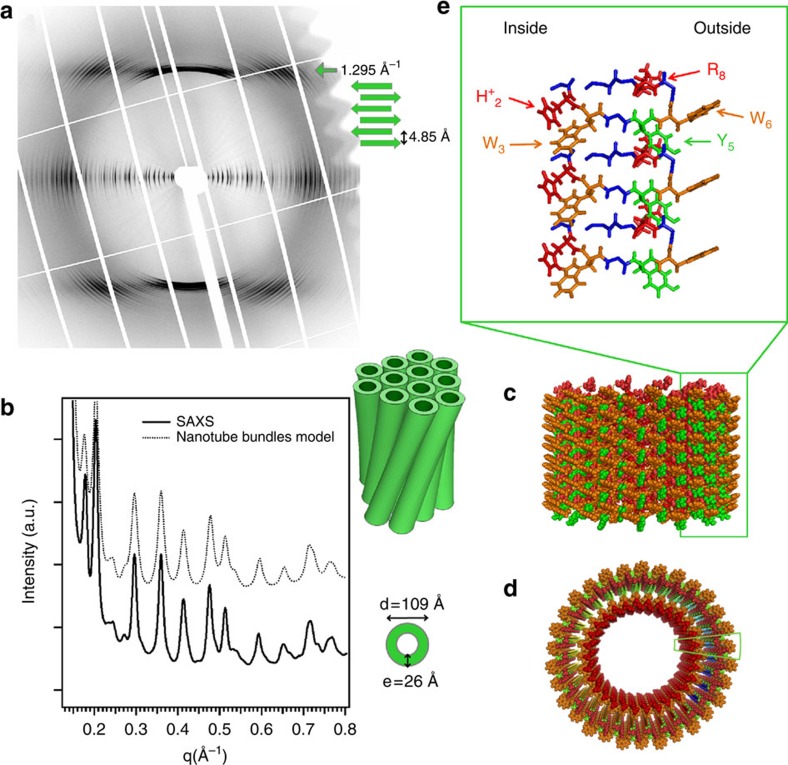
Molecular structure of the low-pH nanotubes. (**a**,**b**) Aligned fibre diffraction (**a**) and SAXS (**b**) patterns of triptorelin bundles of small nanotubes. (**c**) Molecular structure of the protofilaments forming the nanotube walls. Colour code: W (orange), Y (green), protonated histidine (H_2_)^+^ and (R_8_)^+^ (red). (**c**,**d**) Side (**c**) and top (**d**) view of the small nanotubes built from 30 protofilaments. The two green boxes underline the position of one protofilament that is detailed in (**e**).

At low pH, the triptorelin assemblies consist of bundles of regularly twisted small nanotubes (diameter ∼10 nm) (Fig. 1a–d, white arrows on Fig. 1a and zoom on a moiré pattern Fig. 1b and Supplementary Fig. 7 and Supplementary Note 5). Despite the lack of a crystal form at low pH, we could infer a reliable low pH, small nanotube molecular model. To that end, we combined structural information from X-ray diffraction patterns (Fig. 3), from ATR-FTIR and Raman spectra (Fig. 1) and from electron microscopy.

The moire patterns within the bundles indicate a well-defined inter-distance between the nanotubes (Fig. 1a,b). The aligned X-ray fibre pattern (Fig. 3a), with a mosaicity corresponding to the bundle twist, exhibits three well-defined scattering planes, an equatorial one and two layers corresponding to 4.85 Å (ref. 31) (Fig. 3a). The equatorial diffuse scattering is fitted in terms of poly-disperse bundles of nanotubes with a hexagonal packing of 107 Å (Fig. 3b for the model, Supplementary Fig. 8 and Supplementary Note 6). The SAXS peak intensities are in agreement with a wall formed by two shells (Fig. 3b). The nanotube wall full thickness is 26 Å with the outer shell (10 Å) exhibiting a higher electron density than the inner layer. This indicates that there are more aromatic residues located on the outer surface of the nanotube and that they are involved in the close contact between neighbours. The ATR-FTIR spectra show a turn in the peptide structure (Fig. 1i). Consistently with NMR structure determination of Gonadorelin analogues^32^, this turn is likely localized at the level of the (D)W_6_. ATR-FTIR spectra further show an extended antiparallel β-sheet network. The axial position of the 4.85 Å peaks of the X-ray diffraction patterns demonstrates that the β-sheet H-bond network lies along the nanotube axis and forms protofilaments (Fig. 3a). The additional equatorial diffuse scattering at 8–12 Å thus corresponds to the interdistance between these protofilaments. The large number of Bragg peaks in the first layer indicates a very high 3D crystalline sixfold order between nanotubes. This order is only possible with nanotubes that exhibit a six-fold axis, for example, 6n protofilaments per nanotube. Indeed, 30 protofilaments per nanotube yield the correct diameter. The repeat distance along the nanotube axis is the size of the β-hairpin 9.7 Å, for example, 2 × 4.85 Å (Fig. 3c for the structure of the protofilament). The systematic absence of three layers l=2n+1 indicates a centred packing, which corresponds to a 4.85 Å translational shift between each protofilament.

From all this information we built the nanotube molecular model and extract the residue close contacts (Fig. 3c,d, Supplementary Fig. 9 and Supplementary Note 6). In this model, the (D)W_6_ points out of the nanotube. The hexagonal packing of the nanotubes is therefore stabilized by inter-nanotube (D)W_6_ π-stacking as confirmed by Raman spectroscopy. Remarkably, at acid pH, the protonated histidine H_2_ residues are on the inner surface close to W_3_ and in contact with water.

### A key motif of pH-dependent proteins

**Figure 4:**
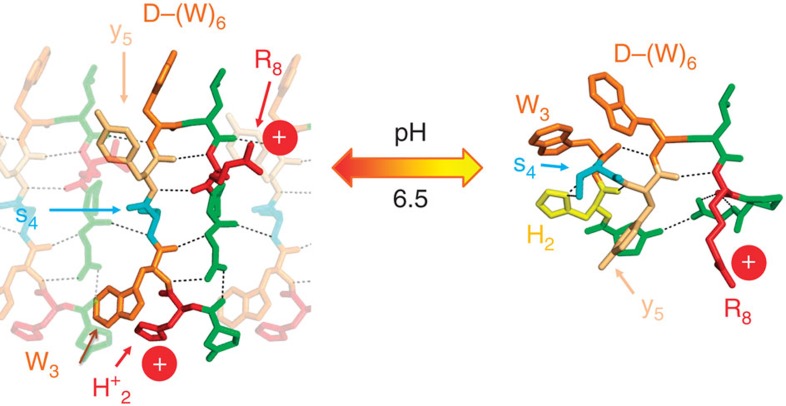
Peptide conformations. Peptide conformation at low (left) and high (right) pH: colour codes: W (orange), Y (light orange), S (cyan), R (red), protonated cationic H (red) and deprotonated neutral and aromatic H (yellow) (Supplementary Movie 1).

The knowledge of both structures that provides the interaction networks stabilizing the high-and low-pH architectures also allows proposing a mechanism of the pH induced diameter change. At high pH, the monocationic peptide with a single-positive charge on R_8_ is coiled at the level of the five contiguous residues H_2_–W_3_–S_4_–Y_5_–(D)W_6_ with five intramolecular interactions among them (Fig. 4, right). At low pH, the repulsive electrostatic forces between the cationic residues, (R_8_)^+^ and (H_2_)^+^ induce an extended conformation of the (H_2_)^+^–W_3_–S_4_–Y_5_–W_6_ sequence of the peptide separating as much as possible the two positive charges on the outer and inner faces of the nanotube wall (Fig. 4, left).

At high pH, H_2_ is involved in an intramolecular interaction network, that is, two strong H-bond H_2_–S_4_ and π– π interactions between H_2_ and Y_5_ (intra- and inter-molecularly) and between H_2_ and W_3_ (Fig. 2e and 4 right). By decreasing the pH, the histidine group protonates (H_2_)^+^ and both H-bond acceptor and aromatic interactions are lost. The coulomb repulsion with (R_8_)^+^ favours the extension of the five contiguous residues H_2_–W_3_–S_4_–Y_5_–(D)W_6_ to seclude (H_2_)^+^ from (R_8_)^+^ inducing a β-hairpin conformation of the peptide. The turn localized around the (D)W_6_ is maintained and the two cations, (R_8_)^+^ and (H_2_)^+^, get close to W_6_ and W_3,_ respectively, forming possible cation-π stabilizing interactions (Fig. 4, left). This large conformational change during the pH jump induces a major packing modification. The well-packed high-pH bilayer essentially maintained by aromatic residues stacking with almost no intramolecular H-bonding (Fig. 2) turns into a typical amyloid like cross-beta organization at low pH (Fig. 3). At high pH, the inner and outer leaflets are quasi-similar so that the wall bending is limited and generates large diameter nanotubes. In contrast, at low pH the high curvature is due to the large steric hindrance mismatch between the two parts of the peptide exposed on the inner and outer faces.

Consequently we here show that the short peptide sequence (H_2_–W_3_–S_4_–Y_5_–W_6_–L_7_–R_8_) folds either as an extended or as a globular conformation depending on pH. The stabilizing forces of the globular folding at high pH come from both tight H-bonding (H_2_–S_4_) and π–π interactions while the repulsive coulomb interaction between the two cations acts as an extender at low pH. These enforce distinct mono- and dicationic peptide conformations that are both hydrophobic enough to self-organize into curved layers, providing two distinct packings into nanotubes exhibiting consequently two distinct diameters (10.7 vs 50 nm), reversibly switchable by pH (Supplementary Fig. 10).

## Discussion

The high efficiency of this pH-induced mechanism of peptide conformational switch suggests biological relevance. We therefore asked whether it might regulate proteins whose function requires large conformational changes precisely triggered by a pH shift. One extensively studied example of such proteins is the diverse families of membrane fusion proteins that enveloped viruses use to enter cells^33^. Many such viral fusion proteins are triggered by the acidification in endosomes after virus internalization. Protonation of histidines has been scrutinized as the triggering mechanism but definite identification of specific pH sensors has been mostly unsuccessful so far^9^,^10^. Strikingly, reanalysis of atomic structures from the PDB and the literature shows that very divergent or even unrelated viral fusion proteins seem to have converged towards combinations of histidine-aromatic clusters and histidine-serine pairs in the key regions that dissociate and/or change structure on lowering of pH (Table 1, Supplementary Fig. 11 and Supplementary Note 7). The evidence for this aromatic-histine-serine switch could be extended to other types of conformationally pH-sensitive proteins. Indeed, the structures of the pH-dependent bacterial membrane channel OmpG^34^ and of the soluble protein M-ficolin^35^ also support this motif as involved in the mechanisms of pH-dependent conformational change (Table 1, Supplementary Fig. 11 and Supplementary Note 7). This non-exhaustive protein structure analysis shows that aromatic pockets and histidine-serine H-bonds may be used by a wide variety of proteins with a biological function that requires a pH-induced conformational switch.

Beyond the biological relevance, this mechanism opens significant downstream perspective in the field of nanomaterials. Indeed, there are very few self-assemblies having well-defined organizations with peptides^20^,^25^,^30^,^36^, proteins^37^ or DNA^11^ and even less that can be continuously tuned^22^,^24^,^38^. One major challenge is the design of systems that can be triggered by external stimuli such as the pH^21^,^39^,^40^. Our example of pH-responsive nanotube diameter is based on a very short peptide sequence of 10 amino acids. The chemical variety of amino acids opens easier and richer functionalization perspectives than other advanced bio-inspired self-assemblies^11^.

In conclusion, we decipher the atomic details of pH-tuned alternate conformations that result in large self-assembly differences. The basic conformation is stabilized by a highly specific histidine-serine H-bond and by histidine-aromatic stacking. The acid conformation is imposed by the repulsive coulomb interactions between both cationic protonated histidine and arginine. This molecular switch is general, as we have found such switches in a large range of proteins including human, viral and bacterial proteins either soluble or insoluble. The conformational molecular switch described here may also be involved in protein folding^41^. Finally, this mechanism opens prospects in the field of nanomaterials where systems that can be reversibly triggered by external stimuli are highly desirable.

## Methods

### Triptorelin peptide

(pE1–H2–W3–S4–Y5–(D)W6–L7–R8–P9–G10–NH_2_) or (pGlu–His–Trp–Ser–Tyr–D–Trp–Leu–Arg–Pro–Gly–NH_2_) or 5-oxo-D-prolyl–L-histidyl–L-tryptophyl–L-seryl–L-tyrosyl–3–(1H–indol–2–yl)–L-alanylleucyl–L-arginyl–L-prolylglycinamide was provided by IPSEN as lyophilized powder and acetate salt (purity >99%; water content 20% and acetate/peptide (MW/MW) ratio is 1.8).

### Raman microscopy (micro–Raman)

Room-temperature Raman spectra were recorded with a Jobin Yvon U1000 spectrometer equipped with a Symphony CCD camera coupled to a BX41 Olympus microscope. Excitation at 514.5 nm was provided by Coherent Argon (Innova 100) laser. No evolution of the resonance Raman signal was observed during data acquisition.

### FT-Raman

Spectra were recorded at 4 cm^−1^ resolution using a Bruker IFS66 interferometer coupled to a Bruker FRA106 Raman module equipped with a continuous Nd: Yag laser providing excitation at 1,064 nm. All spectra were recorded at room temperature with backscattering geometry from samples in hermetically closed glass capillaries. The spectra resulted from 500 to 5,000 cm^−1^.

### ATR-FTIR

ATR-FTIR spectra were recorded at a 4 cm^−1^ resolution with a Bruker IFS 66 spectrophotometer equipped with a 45° N ZnSe ATR attachment. The spectra obtained resulted from the average of 30 scans and were corrected for the linear dependence on the wavelength of the absorption measured by ATR. The water signal was removed by subtraction of pure water spectrum.

### Optical microscopy

Optical microscopy observations were performed using an Axiovert 100 A Olympus inverted microscope, at magnifications up to 20 (A-plan Olympus objectives), on samples conditioned in thin layers between glass coverslips. The polarizer and analyser were in a fixed crossed position. A retardation plate was inserted at 45° to the polarizers. Digital images were taken with an Olympus Highlight 3000 camera connected to a computer.

### Sample preparation for electron microscopy

Negatively stained samples: drops of triptoreline acetate solutions were deposited on formvar-coated electron microscopy grids. After 1 min contact, the excess of water was removed and a drop of 1% uranyl acetate was deposited for 1 min on the top of the sample. The excess of uranyl acetate was removed.

Preparation of the sample for metal replica processing: different techniques for sample fracturing were used: (i) drops of triptoreline acetate solutions were sandwiched between two copper platelets using a 400-mesh gold grid as a spacer. The samples were then frozen at −190 °C by rapid immersion in liquid propane and stored at −196 °C in liquid nitrogen until subsequent use. Frozen samples were fractured by opening the two copper platelets at −150 °C and 2 × 10^−8^ mbar in a Baltec BAF 060 freeze-fracture apparatus (BAL-TEC, Liechtenstein), (ii) drops of triptoreline were directly frozen at −190 °C by rapid immersion in liquid propane and stored at −196 °C in liquid nitrogen until subsequent use. Frozen samples were further fractured by microtomy inside the Baltec BAF 060 freeze-fracture apparatus (BAL-TEC, Liechtenstein). No cryo-protectant was necessary for these preparations.

Freeze-etching was carried out by switching the sample temperature from −150 to −100 °C and maintaining the pressure at 10^−7^ mbar for 2 min to sublimate the surface layer of ice.

Metal replicas of the exposed surfaces (from the different preparations described) were obtained by evaporating 2 nm of platinum with an electron gun at an angle of 45° and 20 nm of carbon with an electron gun at an angle of 90°. After being floated in distilled water, the replicas were picked up on formvar-coated electron microscopy grids.

### Electron microscopy

Either stained samples or freeze-fracture replicas, which were previously deposited on 200-mesh formvar-coated copper grids, were examined with either a JEOL 1,010 or a Philips CM12 transmission electron microscope at 80 kV.

### SAXS, fibre diffraction, X-ray diffraction

SAXS: this was performed on the high brilliance SWING beam line (12 KeV) at the Soleil Synchrotron Facility using sample-detector distances of 0.5–3 m depending on the experiments. The diffraction patterns were therefore recorded for reciprocal spacing *q* (Å^−1^) from 0.007 to 2.1 Å^−1^, that is, a range of repetitive distances *d*=2π/*q* from 990 and 3 Å. The X-ray patterns were detected and recorded via a chip charge-coupled device camera detector, AVIEX. The samples were prepared in 1.3 to 1.6 mm glass capillaries (Glas Technik and Konstruktion, Schönewalde, Germany) and introduced into a homemade capillary holder accommodating 20 capillaries at controlled temperature. All samples exhibited powder diffraction and scattering intensities as a function of the radial wave vector*, q*=4*π* sin(*θ*/*λ*), which was determined by circular integration.

Crystal and fibre X-Ray diffraction were performed on PROXIMA1 (Soleil) beamline using a Pilatus 6 M detector. For crystal diffraction the wavelength was fixed at 0.78471 Å. For fibre diffraction the wavelength was fixed at 0.98011 Å and the detector to sample distance at 0.3202, m. A homemade sample holder adapted built for PX1 beam line maintained the capillary.

### Fibre growth on magnetic field gradient

The bundles of nanotubes obtained from the self-assembly of triptoreline at low pH were grown in a strong magnetic field gradient on the top of a small (1 cm^3^) rare-earth magnets (gift from Sakellariou Dimitrious, DSM/IRAMIS, Cea-Saclay). In this condition, very well-aligned fibres were obtained and analysed by wide-angle X-ray scattering on SWING and PROXIMA1 beamlines (Soleil).

### Triptoreline crystallization

Lyophilized triptoreline was dissolved in water and subjected to standard crystallization screens, by sitting drop and batch methods. Crystallization drops were examined by optical microscopy over times ranging from a few minutes to a few weeks.

Triptoreline monocrystals were obtained in the presence of phosphate buffer. The first crystallization screening shows that small peptide crystals were obtained in a large range of peptide concentration (0.5–8% (w/w)) and of pH buffer (from 6 to 8). To enforce the crystal size growing, we rationalized that we should decrease the crystallization kinetic by working at a pH close to the Histidine pKa. Indeed, the optimized conditions for crystal growth were found at pH 6.2.

More precisely, the monocrystals of triptorelin were obtained when the reservoir contained 500 μl of 300 mM potassium phosphate buffer, pH 6.2 close to the pKa (6.1) determined for the non-assembled peptide (Supplementary Fig. 1b). The drop was a mixture of 10-μl reservoir solution and 10 μl peptide solution (80 mg ml^−1^ triptoreline in water) (4% for the final peptide concentration and 150 mM for the phosphate buffer). The reservoir was filled before the drop. The drop was prepared by quick pipette addition of the peptide solution to the reservoir solution. Crystals were grown at 20 °C. Crystals were transferred to a cryoprotectant (25% glycerol in 300 mM potassium phosphate buffer pH 6.2) before cryogenic processing and data collection.

### Single-crystal structure determination

Ultra-high resolution data were collected to 0.85 Å on Proxima1 beamline at Soleil synchrotron. To reach this resolution, the beam wavelength was adjusted to 0.78471 Å. The X-Ray diffraction pattern recorded for triptorelin mono crystal is shown in Supplementary Fig. 5b. Data integration and scaling were performed using the XDS package642. The structure of triptorelin was solved by dual-space direct methods with SHELXD43. The model was completed by alternative cycles of manual model building with Coot44 and refinement with REMAC545. The data statistics of the triptorelin structure determination are indicated on Supplementary Table 1.

## Additional information

**Accession codes**: Atomic coordinates and structure factors for the reported crystal structures have been deposited with the Protein Data Bank (http://www.pdb.org/) under accession code 4D5M.

**How to cite this article:** Valéry, C. *et al.* Atomic view of the histidine environment stabilizing higher pH conformations of pH-dependent proteins. *Nat. Commun.* 6:7771 doi: 10.1038/ncomms8771 (2015).

## Supplementary Material

Supplementary Information Supplementary Figures 1-11, Supplementary Table 1, Supplementary Notes 1-7 and Supplementary References

Supplementary Movie 1 Peptide conformations

Supplementary Data 1 Atomic coordinates of the low pH nanotube structure

## Figures and Tables

**Table 1:** Examples of pH-sensitive proteins showing histidine-serine H-bonds and histidine-aromatic pockets in the high-pH structures that are absent in the low-pH ones.

**Prot. name PDB ID basic(acid)**	**Protein family**	**Resolution** ***ba*****sic(*****ac*****id)**	**H-bond residues**	**H-bond distances** ***ba*****sic(*****ac*****id)**	**π/π interactions residues**	**π/π interactions distances** ***ba*****sic(*****ac*****id)**
3J27@ (1URZ)	Membrane fusion protein Flavivirus Class II	3.5 Å(2.7 Å)	H_317_*-S_396_*	3.3 Å(14.0 Å)	H_261_-W_19_†H_7_†-W_212_	3.6 Å‡ (N/A†)2.8 Å‡ (N/A†)
3N43(N/A†)	Membrane fusion protein Alphavirus Class II	2.6 Å(N/A†)	H_170_*†-S_57_*	2.9 Å(N/A†)	H_73_†-W_89_	3.6 Å‡ (N/A†)
2J6J(2CMZ)	Membrane fusion protein Rhabdovirus Class III	3 Å(2.4 Å)	H_407_*-S_84_*	3.4 Å(32.2 Å)	H_33_-F_189_	3.9 Å‡ (11.2 Å)
OmpG 2IWW(2IWV)	Bacterial membrane protein	2.3 Å(2.7 Å)	H_231_-S_218_	2.9 Å(9.9 Å)	H_261_-F_233_	4.5 Å (7,9 Å)
M-Ficolin 2JHM(2JHH)	Human soluble protein	1.52 Å(1.7 Å)	H_268_-S_270_H_222_-S_257_	3.5 Å(8.7 Å)3.6 Å(12.7 Å)	H_222_-W_249_H_255_-Y_251_H_255_-Y_283_	3.6 Å (3.3 Å)4.0 Å (10.5 Å)3.6 Å (17.5 Å)

(Supplementary Fig. 11 and Supplementary Note 7)

@ Cryo EM structure of the native virion.

^*^These residues are conserved within each viral genus despite low overall sequence identity.

^†^In the prefusion assembly these interacting residues are harboured by a companion protein that dissociates from the fusion protein on exposure to low pH.

^‡^In all cases of viral fusion proteins, in the prefusion conformation there is at least one histidine-containing aromatic cluster that is conserved (although individual residues may not be) and that is disrupted in the postfusion conformation. The aromatic residue closest to the histidine is given.
