# Supplementary material for: Atomic view of the histidine environment stabilizing higher-pH conformations of pH-dependent proteins
Source: Nat Commun. 2015 Jul 20;6:7771. doi: 10.1038/ncomms8771 (PMC4518280; doi:10.1038/ncomms8771)
Supplement: Supplementary Information — Supplementary Figures 1-11, Supplementary Table 1, Supplementary Notes 1-7 and Supplementary References [file ncomms8771-s1.pdf]

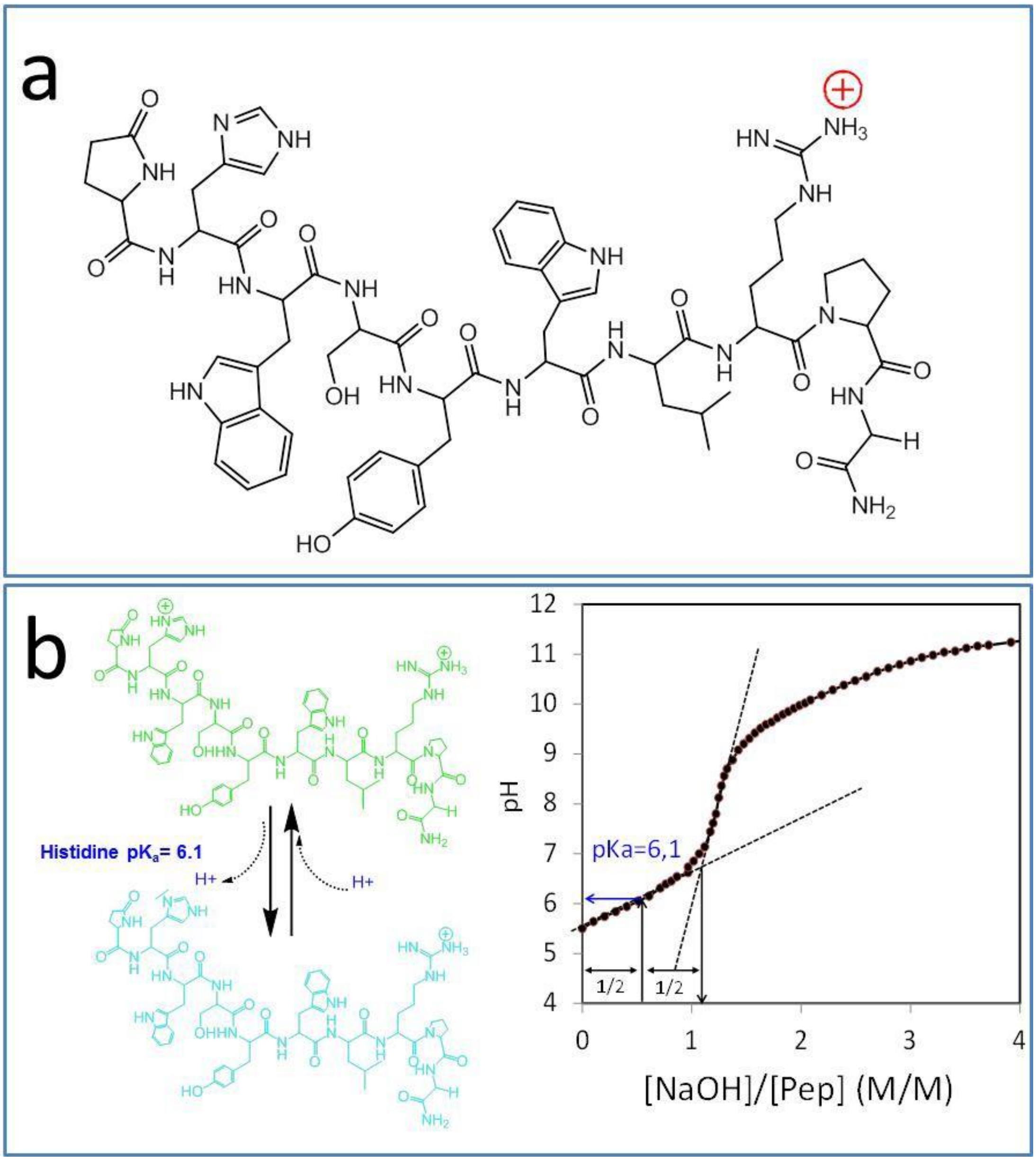

Supplementary Figure 1 : Peptide Sequence And Titration

a : Peptide sequence: (pE$_1$-H$_2$-W$_3$-S$_4$-Y$_5$-(D)W$_6$-L$_7$-R$_8$-P$_9$-G$_{10}$-NH$_2$) or (pGlu-His-Trp-Ser-Tyr-D-Trp-Leu-Arg-Pro-Gly-NH$_2$) and **b:** Peptide titration: Triptorelin is a decapeptide that contains three purely aromatic side chains for pH below 10 (2 tryptophans and 1 tyrosine), one histidine that can be either charged at low pH (below 6.0) or uncharged and aromatic at high pH (pKa = 6.1). Moreover it also contains 1 arginine that remains cationic for a large range of pH (pKa>9) as well as a tyrosine that remains neutral for a large range of pH (pKa>10).

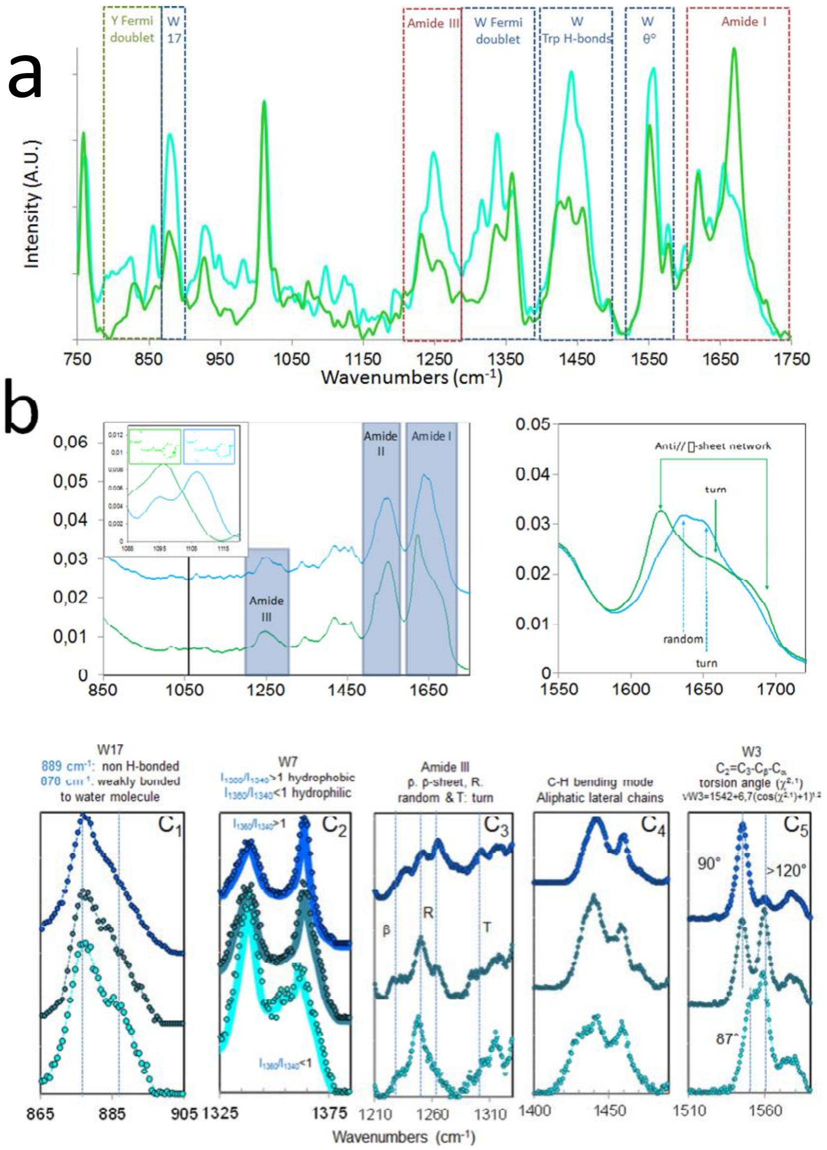

Supplementary Figure 2: Characterization Of Small Nanotubes (Low Ph), Large Nanotubes (High Ph) And Crystals By Vibrational Spectroscopies.

a: FT-Raman spectra of low pH small nanotubes (green) and high pH large nanotubes (blue). On the figure, we have underlined the signals that are characteristic of tryptophan (blue), tyrosine (green) and amide vibration modes (orange) (triptorelin 15% (w/w)).

b, left panel : ATR-FTIR spectra of low pH small nanotubes (green spectrum) and high pH large nanotubes (blue spectra) (triptorelin 5% (w/w)). Amide I, II and III vibration modes are underlined in blue. For more clarity, the spectrum of high pH large nanotubes has been shifted by 0.018 OD. The insert are the region of Histidine vibration modes that depend on its protonation state (see Figure 1 in the article).

b, right panel: The spectra on the right panel are zoomed on the amide I vibration mode.

c: Raman microscopy of large nanotubes (bottom spectrum, 10% (w/w) peptide, pH8.5 adjusted with NaOH), small crystals (middle spectrum) and mono-crystal (top spectrum, from 5% (w/w) peptide at pH 6.2 adjusted with phosphate). Different regions of the spectrum are detailed in panels c_1 to c_5. See supplementary note 4 for explanation.

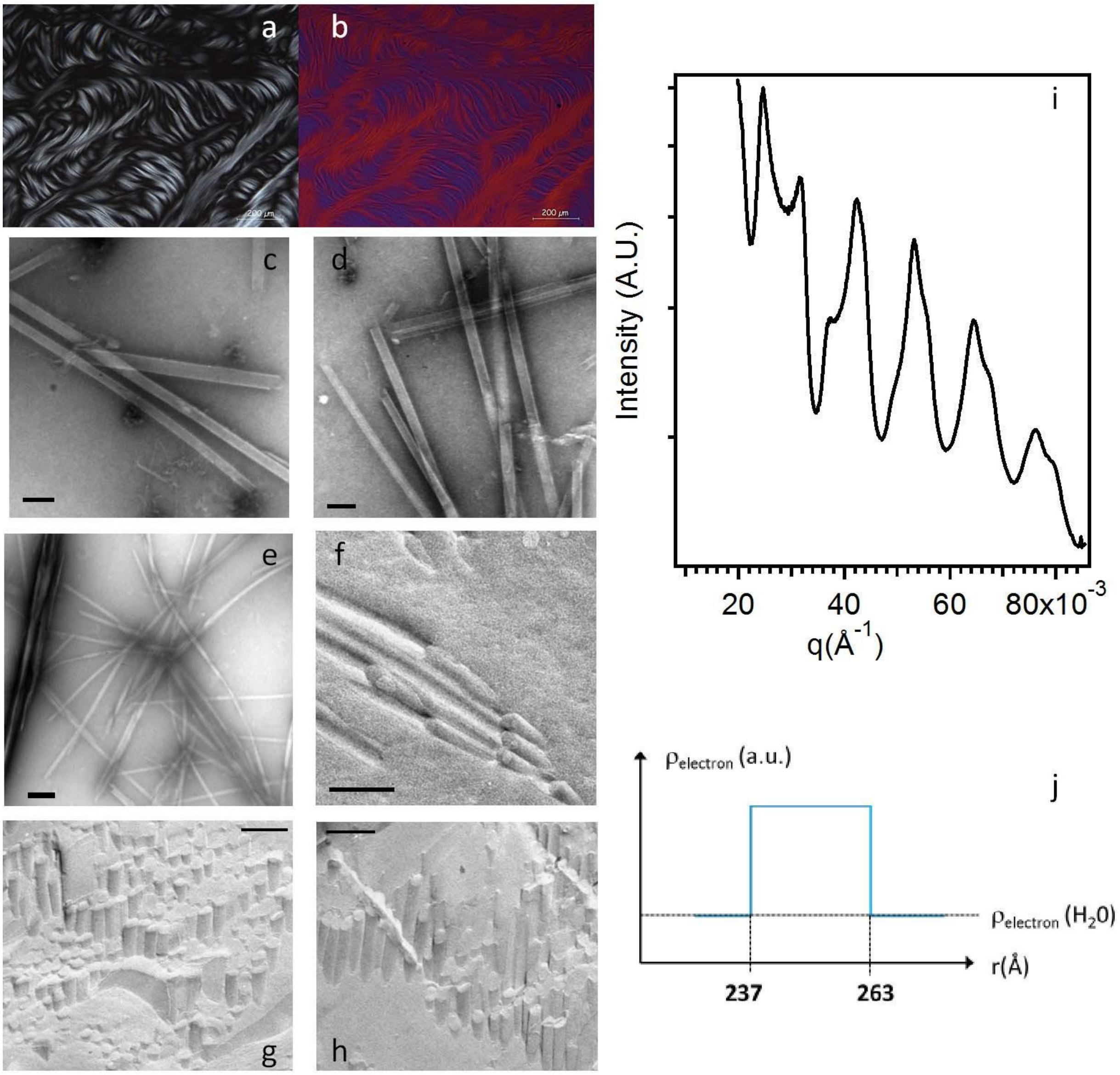

Supplementary Figure 3: Optical Microscopy, Electron Microscopy And Usaxs Of Large Nanotubes

a & b: Triptorelin (5% w/w solution) at pH 8.6 left under polarized light microscopy, and right with a retardation plate samples showing nematic-like textures indicating liquid crystalline structures.
c-f: Electron micrographs of large nanotubes (pH8.5). ***c, d & e*:** images of negatively stained samples; ***f, g & h*:** images of replicas obtained after freeze fracturing and etching of the samples. All the scales are for 200nm except 500nm for Panel **e**.
i: USAXS of high pH large nanotubes showing the hexagonal packing of the nanotubes with the first four Bragg peaks (1, √3, 2,√7) corresponding to a 59nm hexagonal lattice.
j: Radial electron density model of the large nanotube wall and wall thickness.

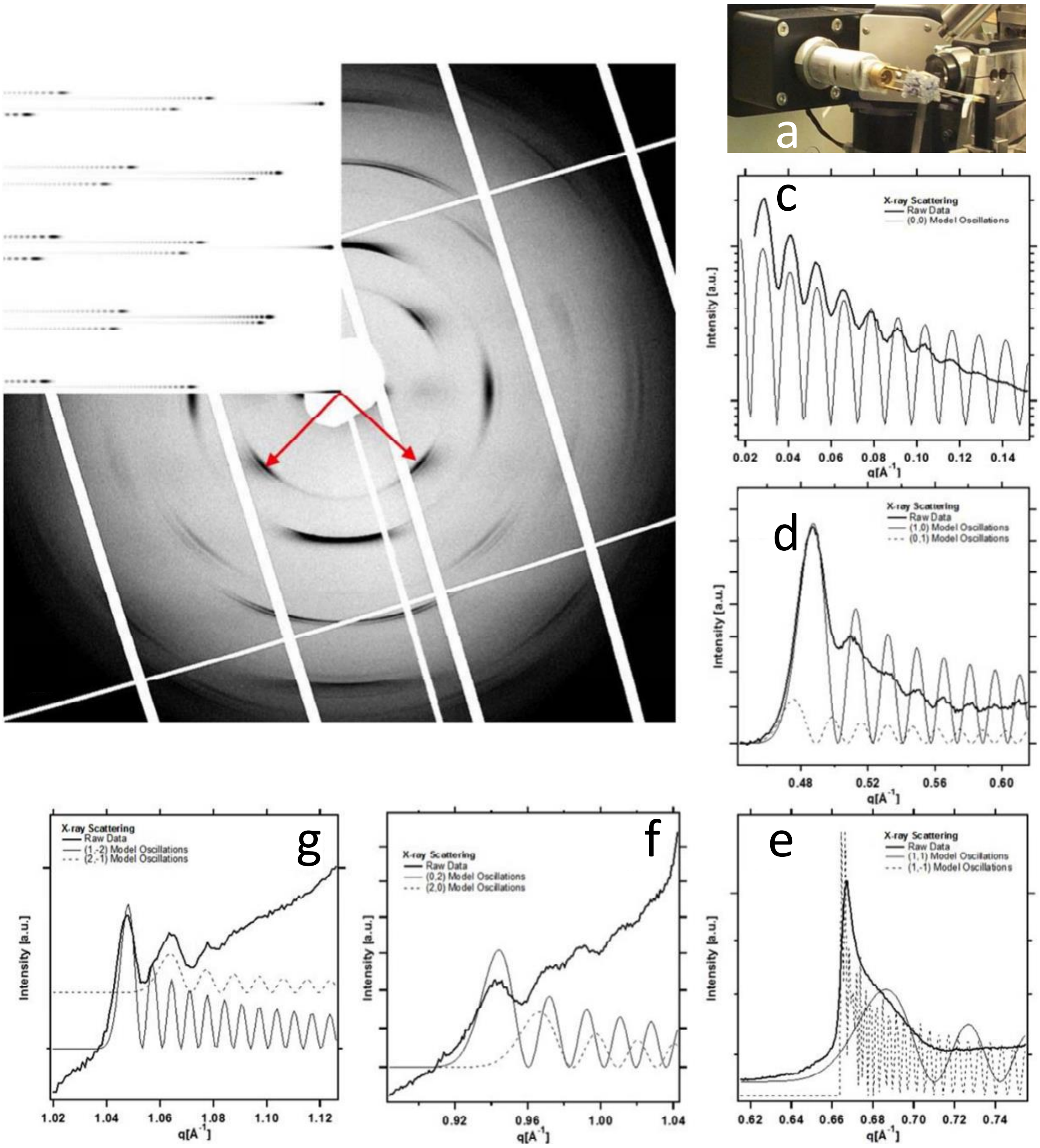

Supplementary Figure 4: Fiber Diffraction And Analysis Of Oriented Large Nanotubes

a: The capillary containing the oriented fibres were maintained by a home made sample holder adapted for PX1 beamline (synchrotron Soleil).
b: X-ray diffraction pattern of aligned high pH large nanotubes together with the theoretical X-ray diffraction pattern. The alignment quality of the sample is good enough to find the indexation of the diffuse scattering. The 2 primary vectors, *i* and *j*, of the 2D crystal are indicated by the two red arrows.
c-g: Powder radial integration patterns and corresponding fits for five selected and representative part of the powder pattern : experimental data (bold lines) and fits (light lines).

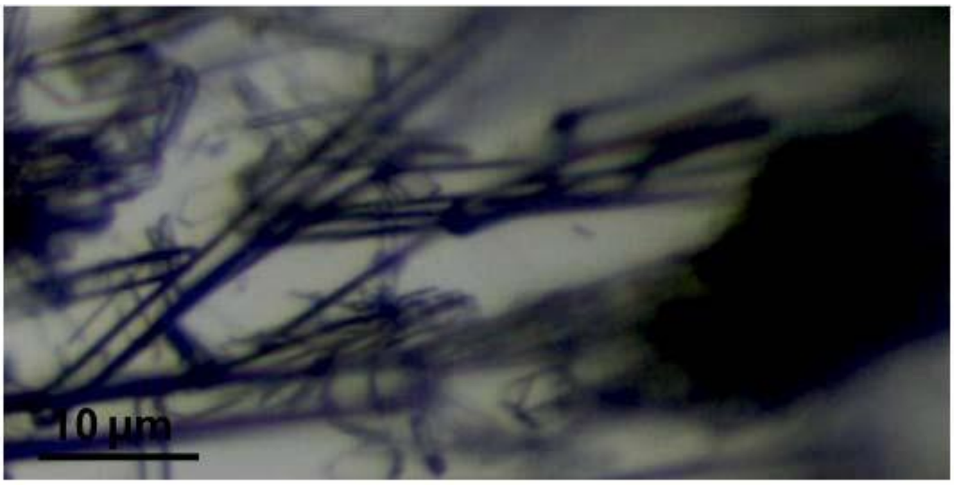

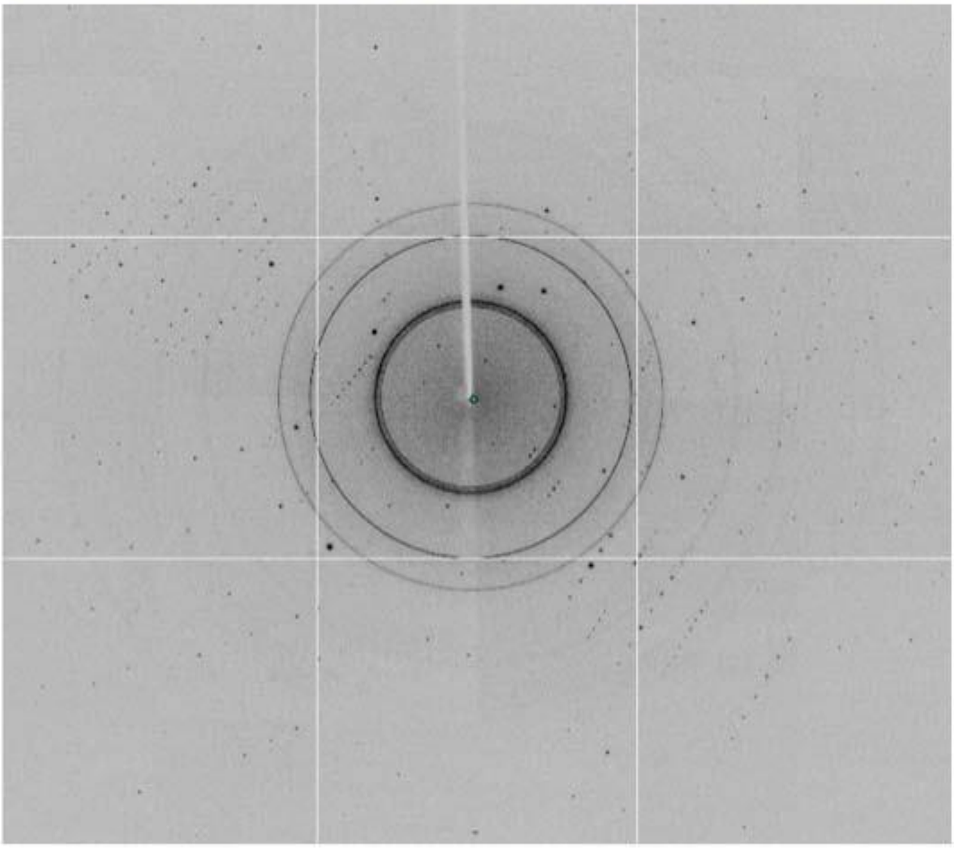

Supplementary Figure 5: Resolution Of The Structure Of Triptorelin Monocrystal

a: Photo of the drop containing the needle shaped crystals of triptorelin phosphate

b: X-Ray diffraction pattern of a Triptorelin monocrystal recorded on PROXIMA1 beamline at SOLEIL synchrotron (France).

Data statistics are on Supplementary Table 1.

Supplementary Figure 6: Stereo Image Of A Portion Of The Electron Density Map Of The Crystallographic Structure

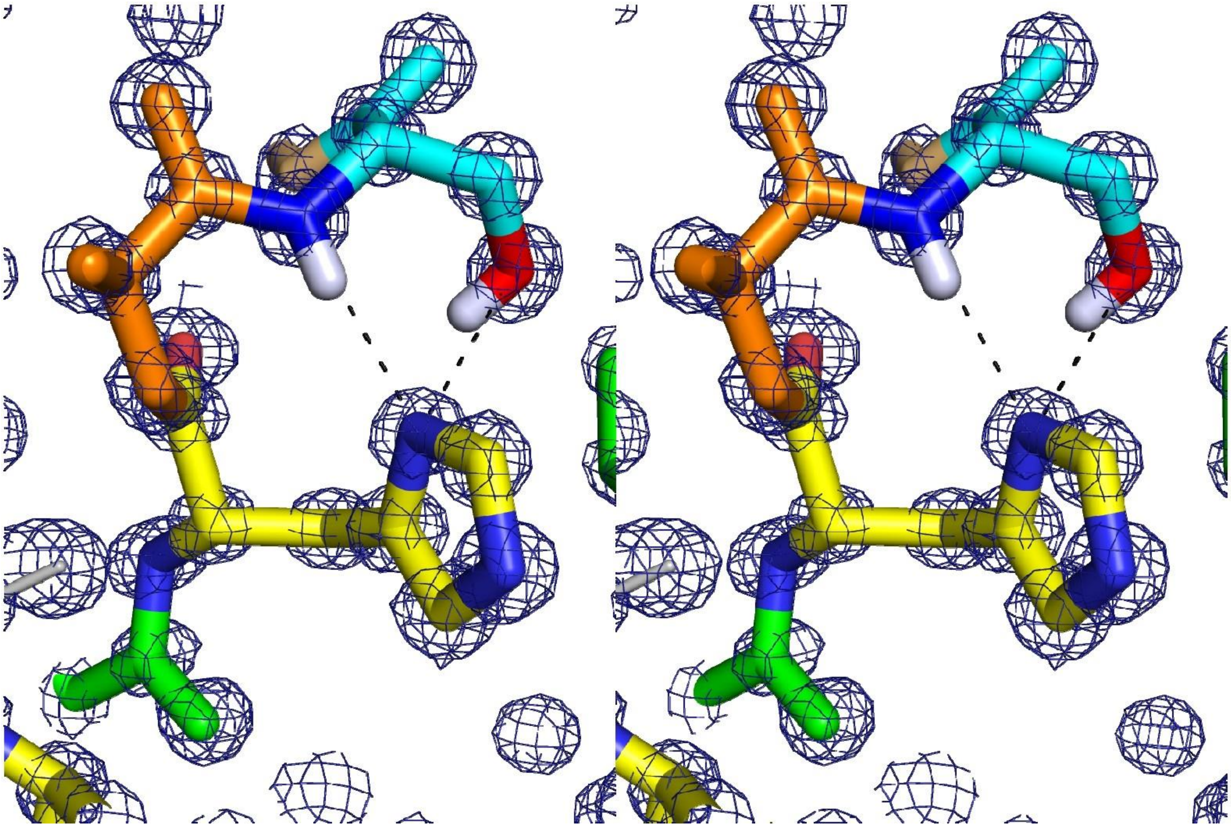

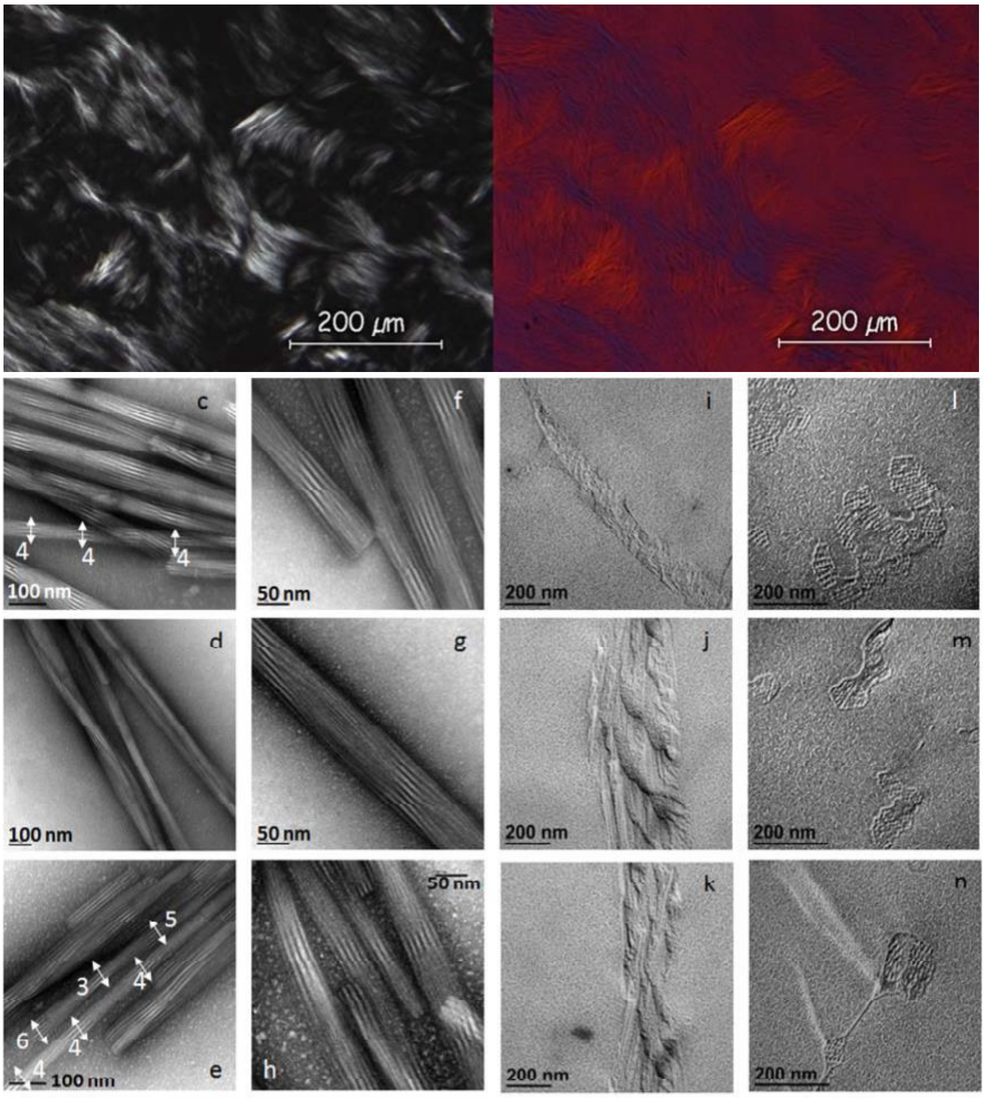

Supplementary Figure 7: Optical And Electron Microscopies Of Small Triptoreline Nanotubes (Low Ph): Characterisation Of Twisted Bundles Of Nanotubes

a & b: Polarized light microscopy on 5% w/w peptide incubated at pH 6 (A), and with a retardation plate (B).

c to h: Electron microscopy of negatively stained (uranyl acetate) small nanotubes. The photos from negative stained samples show moiré pattern indicating that the nanotubes are monodisperse in diameter, perfectly packed in a hexagonal lattice and that they are twisted within the bundles. In the moiré, it is possible to count the number of “filament” like along the axis of the bundles (for example, panel **c** shows a bundle with successively 4,4,4 filament like structures and panel **e,** a bundle with successively 6,3,5 filaments is visible) indicating that the bundles contain different numbers of nanotubes.

i to n: Electron micrographs of metal replicas obtained after freeze fracture of small nanotubes, **i to k**; longitudinal fractures and **l to n** perpendicular fractures. On the photos of the replicas showing perpendicular cuts of the freeze fractured samples we can for example count the number of nanotubes and see the polydispersity.

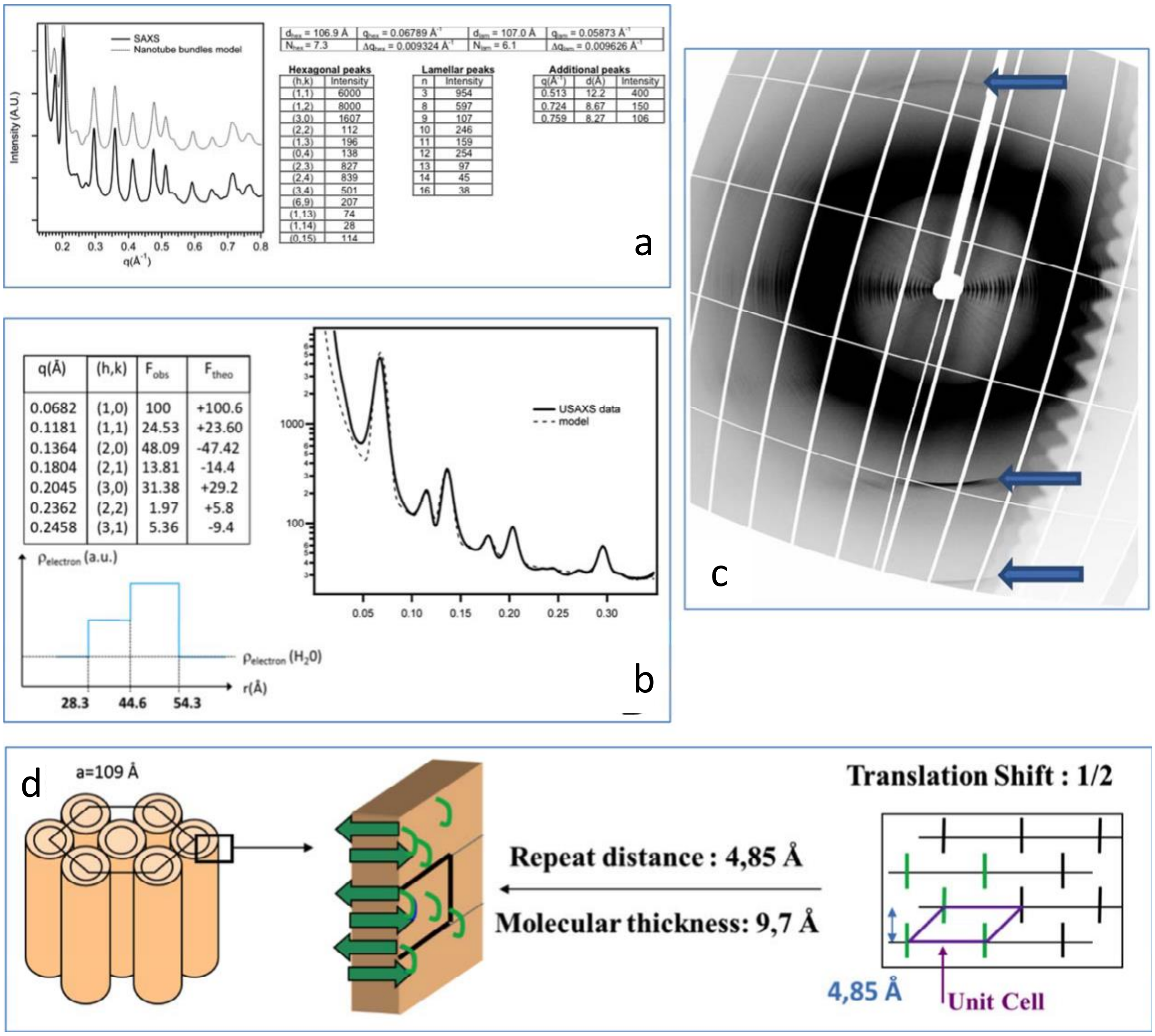

Supplementary Figure 8: Usaxs And Saxs Data And Treatment, Diffraction Pattern Of Aligned Small Nanotubes And Starting Model For The Peptide Packing With The Nanotube Wall.

a: SAXS pattern of small nanotube bundles (plain line) and nanotube bundle model (dotted line). The fit parameters for the nanotube bundle model are reported on the table

b: USAXS pattern (plain line) and model (dotted line). The comparison of the experimental and the fit data are summarized on the Table. The electron density variation of the nanotubes is shown on the sketch at the bottom of panel B: the inner radius is 28.3Å and the outer radius is 54.3 Å.

c: Oversaturated fiber diffraction pattern of an aligned sample revealing the second and third order of the beta-sheet (blue arrows).

d: Scheme of the small nanotube bundles from the supramolecular (hexagonal lattice of small nanotubes (left) to the molecular packing of the peptides within the wall (intermediate and right scheme).

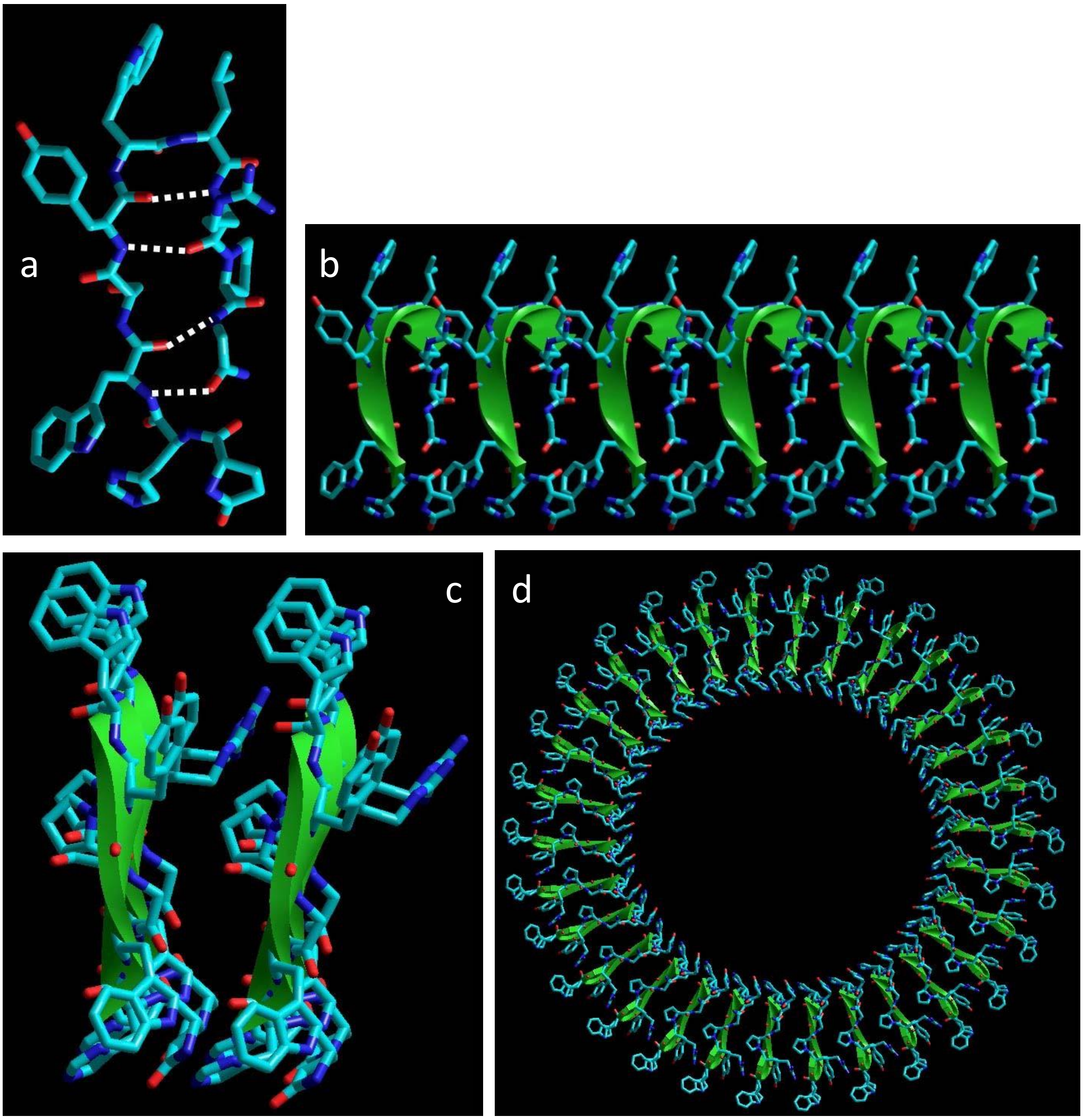

Supplementary Figure 9: The Molecular Model Of The Small Nanotubes: The Steps And The Final Assembly.

a: Step 1: Construction of the β-hairpin conformation of the peptide.

b: Step 2: Construction of the β-sheet protofilament (side view).

c: Step 3: Construction of two close β-sheet protofilaments (top view).

d: step3X30: Construction of a full small nanotube formed by 30 protofilaments with a rotation angle of 12° between each protofilaments (top view).

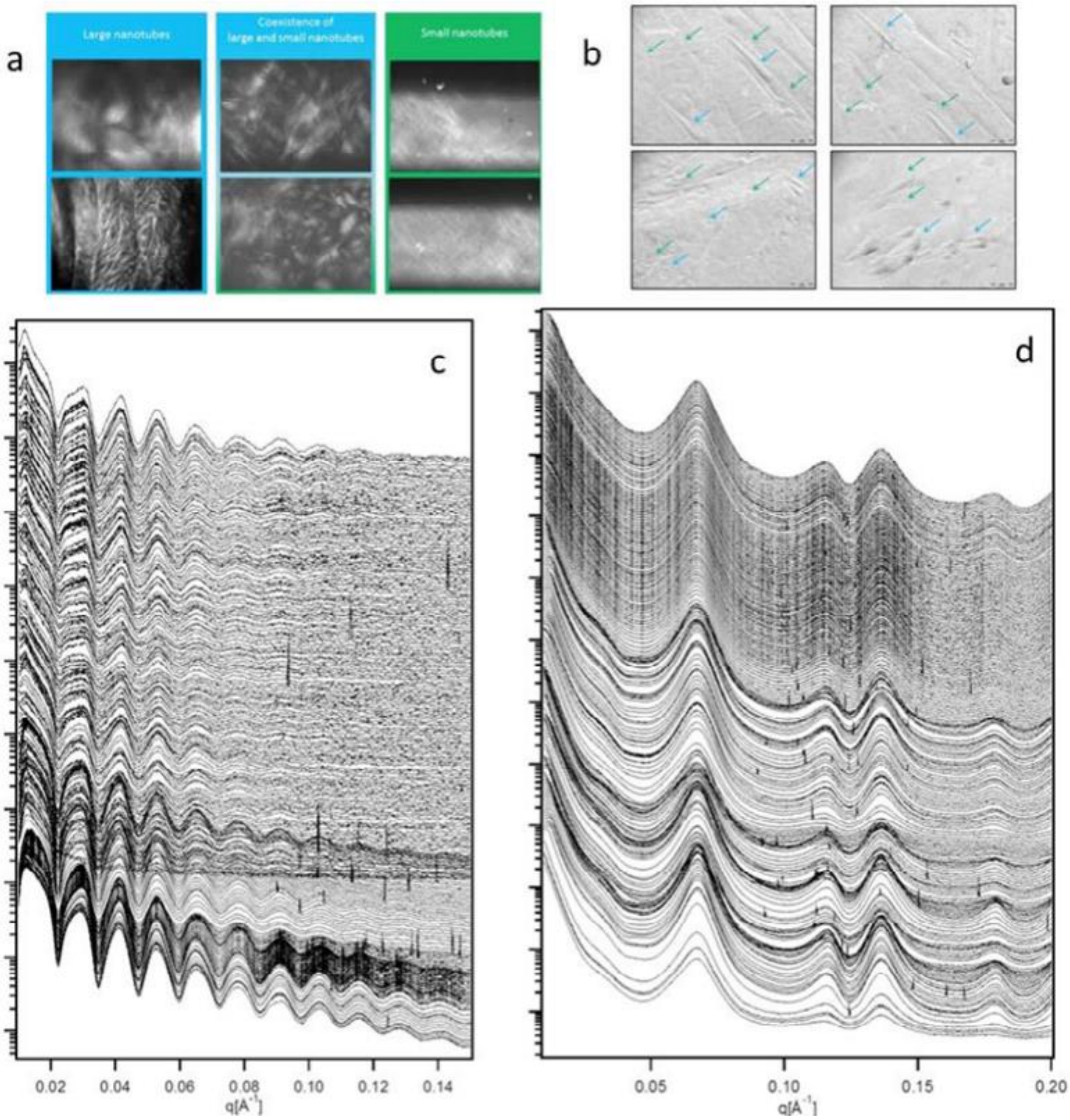

Supplementary Figure 10: Small Nanotubes And Large Nanotubes: Coexistence And Transitions.

a: Coexistence of large and small nanotubes observed by polarized light microscopy

b: Coexistence of large (blue arrows) and bundles of small nanotubes (green arrows) by electron microscopy after freeze fracture

c: The transition from small to large nanotubes. SAXS patterns recorded from the bottom to the top (2cm) of a capillary that initially contains an excess of large nanotubes on the bottom of the capillary and bundles of small nanotubes on the top of the capillary in contact with the large nanotubes. After few days of equilibration, all the capillary contains only large nanotubes

d: The transition from large to small nanotubes. SAXS patterns recorded from the bottom to the top of a capillary (2cm) that initially contains an excess of bundles of small nanotubes on the bottom of the capillary and large nanotubes on the top of the capillary in contact with the bundles of small nanotubes. After, few days of equilibration, all the capillary contains bundles of small nanotubes

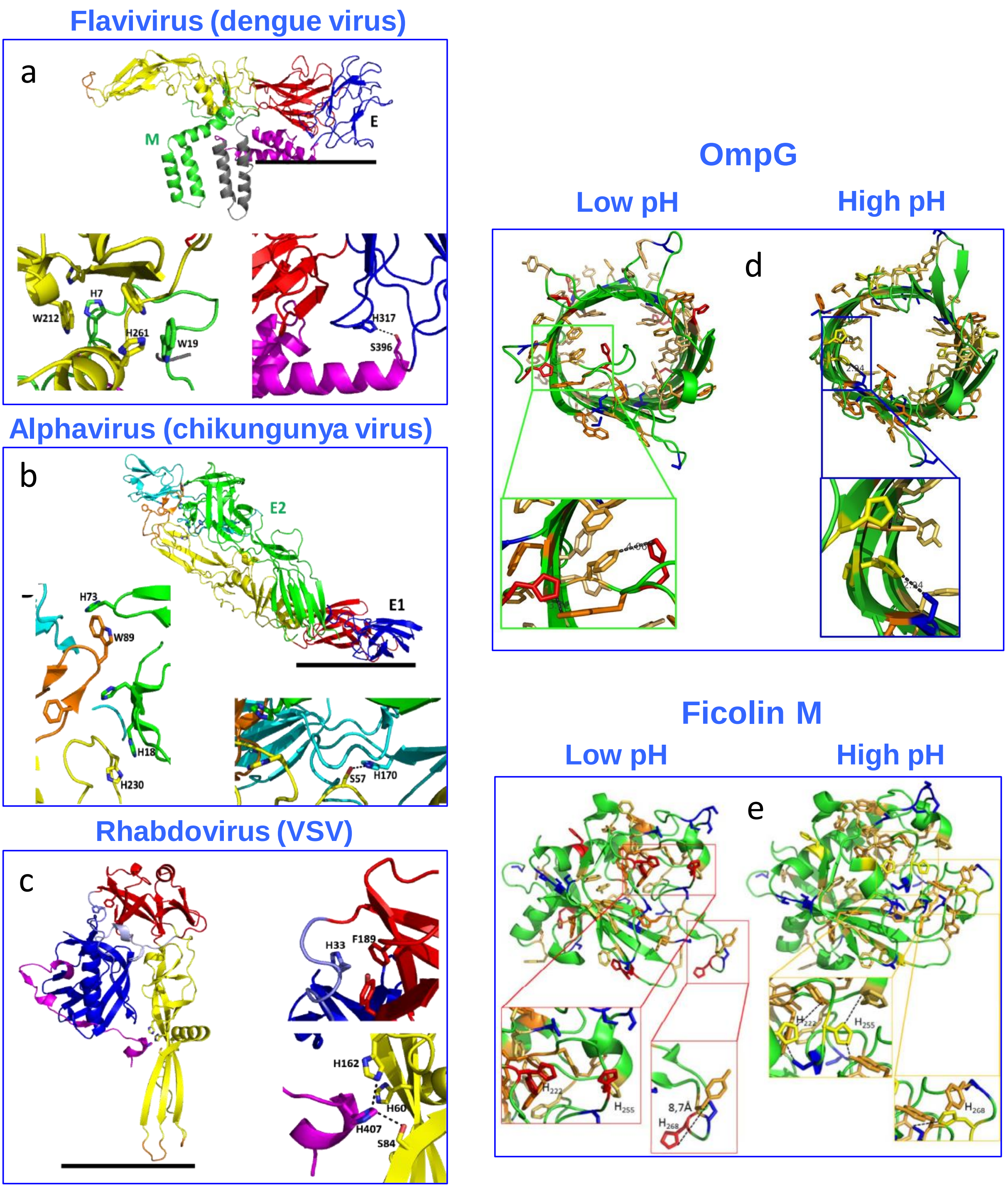

Supplementary Figure 11: Generality Of Histidine-Serine And Histidine-Aromatics Conformational Switches

a: The flavivirus pre-fusion (high pH) heterodimer E/M (dengue virus, pdb code: 3J27; chains C and D). The class II fusion protein E is colored by domains as explained in the text. The companion protein M is in green. **Upper scheme**: overall view of the E/M heterodimer, the horizontal black line indicates the approximate start of the viral membrane. **Lower and left**

scheme: close up of the main region of E/M interaction, that must break for E/M dissociation. Conserved residues are displayed as sticks and include histidines in an aromatic environment, as noted[1]. **Lower and right scheme**: close up of the hinge between the lateral domain (blue) and stem (magenta), both of which undergo major relocation in the post-fusion trimer[25].
b: The alphavirus pre-fusion (high pH) heterodimer E1/E2 (chikungunya virus, pdb code: 3N43; chains F and B[2]. The class II fusion protein E1 is colored by domains as explained in the text. The companion protein E2 is in green and cyan. **Upper scheme**: overall view of the E1/E2 heterodimer, the horizontal black line indicates the approximate start of the viral membrane; **Lower left scheme**: close up of the region where E2 caps the fusion loop of E1. Conserved residues are displayed as sticks; **Lower right scheme**: close up of the location of the conserved strong H-bond between S_{57} of E1 and H_{170} of E2. The part of E2 depicted in cyan, including H170, dissociates from E1 at low pH[3].
c: The single rhabdovirus envelope protein G (class III) in its pre-fusion (high pH) conformation (vesicular stomatitis virus, pdb code: 2J6J[4]). G is colored by domains as explained in the text. **Left scheme:** overall view of the G monomer, the horizontal black line indicates the approximate start of the viral membrane. **Higher right scheme**: close up of the major hinge region. A previously unnoticed cluster of aromatic amino acids including a single histidine is displayed as sticks. **Lower right scheme**: close up of the cluster of conserved histidines previously reported as a pH-dependent switch[26].
d: OMPG protein pH-dependant conformational switch. Crystal structures of OmpG[34] at acidic pH (**left scheme**; 2IWV (2.7Å resolution)) and basic pH (**right scheme**); pdb codes: 2IWW (2.3Å resolution). Top view of the OmpG proton channel and Zoom on the switching domains.
e: M-Ficolin protein pH-dependant conformational switch[35]. **Left scheme:** structure at acid pH (pdb code: 2JHH) and **right scheme** (pdb code 2JHM): structure at high pH. Zooms on hydrogen-bonded His 268 in the basic form (right panel, distance His 268 – Ser 270 of 3.5Å), versus non-hydrogen bonded His 268 in the acidic form (left panel, distance His 268– Ser 270 of 8.7 Å). Moreover, the His 222 and His 255 switches from one aromatic (neutral pH) to a another aromatic (low pH) environment, probably changing from a p-stacking type interaction (high pH) to a cation-p type interactions at low pH.

Supplementary Table 1: Crystal structure, Data collection and refinement statistics

	Triptorelin PDB 4D5M
**Data collection**	
Space group	*C2*
Cell dimensions	
a, *b*, *c* (Å)	84.9, 27.5, 13.1
α, β, γ (°)	90, 98.83, 90
Resolution (Å)	42-0.85 (0.87-0.85)
R_{merge}[a]	0.070 (0.204)
$I / \sigma I$	25.6(5.4)
Completeness (%)	98.6(88.0)
Redundancy	26.6(6.2)
**Refinement**	
Resolution (Å)	42-0.85 (0.87-0.85)
No. reflections	24,802
R_{work} / R_{free}	0.081/0.086 (0.175/0.163)
No. atoms[b]	441
Protein[b]	380
Ligand/ion	18
Water[b]	43
B-factors	1.7
Protein	1.5
Ligand/ion	2.0
Water	4.6
R.m.s deviations	
Bond lengths (Å)	0.015
Bond angles (°)	1.63

Values in parentheses are for highest-resolution shell.
[a] Five datasets collected from one crystal where merged. [b] Omitting hydrogen atoms.

[AU: Wavelength of data collection, temperature and beamline should all be in Methods section.]

Supplementary note 1 : Conformations of triptorelin into the different low and high pH assemblies probed by Raman and ATR-FTIR spectroscopies.

In between the self-assemblies at low (Supplementary Fig. 2a: green spectrum) and high (Supplementary Fig. 2a: blue spectrum), the FT-Raman spectra show large differences in the secondary structure of the peptide (Amide I and III vibration modes), in the environment (W Fermi doublet and W H-bonds bands especially) and conformation (W torsion angle) of the two tryptophan side chains and in the environment of the tyrosine side chain (Y Fermi doublet).

Concerning the signals associated to W, they are difficult to interpret because of the presence of two identical side chains. For example, the W Fermi doublet indicates a globally hydrophobic environment for the W in the small tubes (low pH) but at high pH, the W Fermi doublet shows a doublet of doublets indicating that the two W have different environments. Similarly, the W torsion angle and W H-bond bands show that the two W in the large tubes are not equivalent. However, these experiments do not allow the attribution of the signal to a precise W in the sequence but demonstrate the key changes in the environment of the two W between the small (low pH) and large (high pH) nanotubes.

Concerning the secondary structure (Amide I and III- Supplementary Fig. 2a for FT-Raman and Supplementary Fig. 2b for ATR-FTIR), the peptide in the two self-assemblies does not have the same H-bond network as shown for example by the large differences of the spectra in the Amide I region of the Raman spectra. These differences are underlined on the ATR-FTIR spectra zoomed on the Amide I (Supplementary Fig. 2b, right panel). The Amide vibrations are mainly due to stretching vibrational modes of the peptide backbone. These vibrations are conformational markers through their correlation with hydrogen bonds, in which the backbone carbonyls (Amide I) and NH (Amide II and III) are involved[5,6]. The ATR-FTIR spectrum of the low pH small nanotubes (Supplementary Fig. 2b, right panel and green line) shows three vibrations that can be assigned to β-sheet organization (1620cm^{-1} and 1680-1693cm^{-1}) and turn secondary structure (1657cm^{-1}). On the ATR-FTIR spectrum of high pH large nanotubes (Supplementary Fig. 2b, right panel and blue line), the two sets of vibrations at 1620cm^{-1} and 1680-1693cm^{-1} assigned to the antiparallel β-sheet network almost disappear in favour to a strong vibration at 1640cm^{-1}. This vibration can be assigned to random conformation. The turn secondary structure (1658 cm^{-1}) is present in both low pH small nanotubes and high pH large nanotubes.

These Raman and ATR-FTIR spectra are the signature of the interaction networks that are created in the peptide small low pH and large high pH nanotubes.

Supplementary note 2: Structural analysis of high pH nanotubes

SAXS Analysis of the large nanotubes: hexagonal lattice, nanotube diameter and wall thickness.

The USAXS pattern of high pH large nanotubes shows the hexagonal packing of the nanotubes with the first four Bragg peaks (1, √3, 2,√7) that corresponds to a 59nm hexagonal lattice (Supplementary Fig. 3i). This data allow the construction of the radial electron density model of the large nanotube wall and wall thickness (Supplementary Fig. 3j).

WAXS Analysis of the large nanotubes: determination of the molecular packing

The WAXS data were recorded on PX1 beam line (synchrotron Soleil) using a homemade capillary holder as shown on Supplementary Fig. 4a. The X-ray diffraction pattern of aligned high pH large nanotubes is good enough to find the indexation of the diffuse scattering

(Supplementary Fig. 4b). The analysis in terms of Bessel functions[7] is not possible directly from the 2D fiber pattern (Supplementary Fig. 4b) but possible after fitting of the powder radial integration pattern (Supplementary Fig. 4c to 4g).

The thin nanotube is a curved 2D crystal with two primary vectors (Supplementary Fig. 4b, red arrows). These two primary vectors define two classes of filaments along **i** (resp. **j**) with a width defined by ***j*** (resp. ***i***). The nanotube can be defined by the lateral association of *n* (resp. *m*) filaments. The nanotube exhibits a *n*-fold and a *m*-fold symmetry. Each diffuse scattering is indexed as (*h*,*k*) and corresponds to the position of a "2D"-diffraction peaks $h.\mathbf{i}^* + k.\mathbf{j}^*$. In the reciprocal space, the X-ray scattering is a horizontal line parallel to the equator at $\mathbf{q_z}^* = h.\mathbf{i_z}^* + k.\mathbf{j_z}^*$. The radial profile $I(q_r)$ is a function of the Bessel Function $J_l\,(q_r.r°)$ of order $l=h.n+k.m$ with $r°$ the mean diameter of the nanotubes[15,17].

The knowledge of *n*, *m*, $\mathbf{i_z}^*$, $\mathbf{j_z}^*$ and $r°$ are sufficient to calculate the main oscillations of the 2D X-ray fibre pattern. The powder radial integration can be then calculated and compared to the raw experimental data. All (*n*,*m*) couple solutions have been evaluated by optimization of $\mathbf{i_z}^*$, $\mathbf{j_z}^*$. Only one (*n*,*m*) couple solution is in agreement with the five observed X-ray scattering (Supplementary Fig. 4C to H).

$\mathbf{i_z}^*$	*n*	$\mathbf{j_z}^*$	*m*	r°
0.3425	79	-0.321	-88	250Å

The theoretical 2D X-ray pattern is superimposed on the top-left corner of the 2D experimental X-ray pattern (Supplementary Fig. 4b)

The mean quasi-2D crystal parameters can be extracted from the Bessel analysis. The unit cell parameters are 13.35Å, 12.93Å and 88.47°. They are very similar to the single crystal parameters *b*/2 = 13.75Å, *c* = 13.1Å and γ = 90° (Supplementary Table1). This confirms the Raman analysis that shows similar packing in both high pH nanotubes and single crystal.

Supplementary note 3: Crystal structure determination

Ultra-high resolution data were collected to 0.85 Å on Proxima1 Beamline at Soleil synchrotron. In order to reach this resolution, the beam wavelength was adjusted to 0.78471 Å. The X-Ray diffraction pattern recorded for triptorelin monocrystal is shown on Figure S14. Data integration and scaling were performed using the XDS package6[8]. The structure of triptorelin was solved by dual-space direct methods with SHELXD[9]. The model was completed by alternative cycles of manual model building with Coot[10] and refinement with REMAC5[11].

Supplementary note 4: Comparison of the Raman spectra of the high pH nanotubes, small and large crystals

The micro-Raman technic allows focusing the beam on a particular object for recording the spectra. In the case of small and large crystal, the spectra essentially come from the crystals without the solution (for the small crystals the measurements are done on a crystal pellet). In the case of the gel that contains large nanotubes, the spectrum arises from both the assembled peptide and the solution. Indeed, these objects are in equilibrium with fully hydrated.non-assembled peptides

Supplementary Fig. 2c detailed different Raman signals related to W, CH- bending of lateral chain or amide III recorded for large nanotube (bottom and cyan spectrum), small crystals (middle and dark green spectrum) and large crystal (top and bright blue spectrum:). Some of these signals are well characterized Raman vibration modes that can be interpreted in terms of structure and conformation.

- The νW17 vibration (865-905 cm^{-1}) is an indicator of indole N-H hydrogen bonding[12] (Supplementary Fig. $2c_1$). The three spectra present a similar asymmetric shape indicating the presence of two peaks at 889 cm^{-1} and 878 cm^{-1}. According to Takeuchi (2003)[12], these peaks can be attributed, respectively to a non H-bond W and to a W weakly bonded to a water molecule. The similarity of the three spectra indicates that in the three different samples, the two W of the peptide are in two different environments. We can also notice that for the large nanotubes the contribution of H-bond W is larger than for the crystal spectra. We believe that this difference comes from the non-assembled and fully hydrated peptide recorded together with the large nanotube spectrum.

- The doublet at 1360 cm^{-1} and 1340 cm^{-1} (νW7 doublet, Supplementary Fig. $2c_2$) is due to a Fermi resonance between a fundamental in plane vibration, with one or more combination bands of out-of-plane vibrations[12,13]. The intensity ratio I_{1360}/I_{1340} reflects a hydrophobic ($I_{1360}/I_{1340}>1$) or a hydrophilic ($I_{1360}/I_{1340}<1$) environment of the Tryptophan side chain. Obviously from large nanotubes to large crystal, the environment of both Trp become more and more hydrophobic as seen by the increase of I_{1360}/I_{1340} going from 0.7 (large nanotubes) to 1.2 large crystals. This can easily be understood by considering that the crystals are formed by the tight lamellar packing of the wall of the large nanotubes. In the last case the wall is in contact with bulk water whereas within the crystal, the interlayer water is very small and surely not bulk water. We again have also to consider that for the nanotube, the non-assembled peptide also contributes to the spectrum.

- The Amide vibrations can be use as conformational markers through their correlation with hydrogen bonds in which the carbonyls and N-H are involved. On Supplementary Fig. $2c_3$ we plot the Amide III vibration (1210-1320cm^{-1}) of the peptide[27]. On the three spectra plotted on Supplementary Fig. 2c3, and in agreement with the ATR-FTIR spectra in Figure 2a, almost no β-sheet is detected for the three different samples. The peaks between 1250 and 1260 cm^{-1} can be attributed to random conformation the weak peak at about 1300-1310 cm^{-1} to the turn conformation. From nanotubes to large crystals the Amide III spectra from broad becomes narrower indicating an increase of the constraints on the peptide backbone as expected for the peptide in a crystal packing.

- The vibration bands between 1400 and 1500 cm^{-1} (Supplementary Fig. $2c_4$) are a combination of νW6 mode and C-H bending bands of aliphatic side chains[8] and cannot be interpreted directly in terms of conformation or environment. However, the similarity of this part of the spectra for the three different organizations is in favor to a similar environment of the peptide.

- The 1550 cm^{-1} Raman Tryptophan vibration (νW3 vibration, Supplementary Fig. $2c_5$) is a conformational marker mainly contributed by the indole C_2=C_3 stretch. The W3 wavenumber is affected by the tryptophan torsion angle ($\chi^{2,1}$), which can be approximated by previously established equations[14]. For this part of the spectra and in agreement with νW17 spectral shape, the two Trp of the peptide are in two different environments as shown by the W3 vibration mode doublet. Moreover, this doublet is detected independently of the type of sample. If the vibration at 1560cm-1 (indicative of a torsion angle larger than 120°) is constant over the three samples, the one at lower wavenumbers shifts from 1550 cm^{-1} (large nanotubes) to 1545 cm^{-1} (small and large crystals) indicating a slight change in the torsion angle from 85° to 90° and therefore an increasing constraint on the Trp cycle from the nanotubes to the crystals. For the large crystals, the peak at 1560cm^{-1} still exists but its intensity decreased compared to the two other spectra. In Raman spectroscopy, it is difficult to rely on the intensities, whereas the position of the peaks is more informative. The decrease of this intensity can be due to symmetry reasons within the large crystal.

As a conclusion, these micro-Raman spectra indicate that for all the three samples, the two Trp of the peptide sequence are in two distinct environments in terms of hydrophilicity/hydrophobicity, H-bonds and structural constraints. However, for the large nanotubes, the Trp are in a more hydrophilic environment compared to small or large crystals. The amide III vibrations indicate the absence of β-sheet for all the samples. Finally, the Raman spectra of crystals show narrower signals indicative of more constraints on the peptide conformation as it is expected for a peptide in a crystal packing.

Supplementary note 5: Optical and electron microscopies of small nanotubes

Under polarized light, birefringent regions arising from anisotropic domains (Supplementary Fig. 7a) were observed. Observation using a retardation plate, (Supplementary Fig. 7b), confirmed that these anisotropic domains are due to specific liquid crystalline defects (observation of continuous orientations variations of the slow (blue) and fast (orange) optical axes of the birefringent objects).

The series of electron micrographs on Supplementary Fig. 7c-n show the twisted bundles of nanotubes either from negatively stained samples (c to h) or after freeze-fractures and metal replicas (I to n). From these photos we could see that very often the twisted bundles of nanotubes are associated to other bundles.

The photos from negative stained samples show moiré pattern indicating that the nanotubes are monodisperse in diameter and that they are twisted within the bundles. In the moiré, it is possible to count the number of “filament” like along the axis of the bundles (for example, Supplementary Fig. 7c shows a bundle with successively 4,4,4 filament like structures and Supplementary Fig. 7e a bundle with successively 6,3,5 filaments is visible) indicating that the bundles contain different numbers of nanotubes. These observations are consistent with the photos replicas showing perpendicular cut of the freeze fractured samples (Supplementary Fig. 7i, m & n) on which we can count the number of nanotubes and see the polydispersity of the bundle size.

Supplementary note 6: Structural analysis of low pH bundles of small nanotubes

To get supplementary structural information we prepared aligned sample for SAXS and WAXS. We took advantages of the slow self-assembly kinetic of triptorelin in water and let grow the small nanotubes bundles in a magnetic gradient. Small X-ray capillaries were filled out with the peptide solution and left few days in contact with rare earth magnets. At the surface of the capillary, big individual fibres were pointing out of the solution. The WAXS data were recorded at the extremity of such fibre.

SAXS and USAXS Analysis: radial profile at low resolution

The SAXS and USAXS patterns and their fits are presented on Supplementary Fig. 8, panels a and b respectively.

Electron micrographs showed that the nanotube bundles are polydisperse in terms of size, *i.e.* number of nanotubes per bundle (Supplementary Fig. 7c to n). Due to this polydispersity, there is no full X-ray Scattering model available that fits our data. The SAXS patterns of all the ideal cases have been calculated in the Fibre Diffraction reference book[15]. It predicts the broadening of the hexagonal peaks as well as the enhancement of pseudo lamellar peaks that are due to the similar positions of J_n Bessel functions maxima.

In the present case, the SAXS pattern is fitted by Lorentzian peaks with position of the hexagonal lattice for the first Bragg peaks and only “Bessel” maxima at wider angles. The widths are identical for all hexagonal peaks and correspond to 7.3 nanotubes large bundles. The widths are identical for all lamellar peaks and correspond to 6.1 layers large bundles. Three additional peaks are observed and attributed to the distance between protofilaments. Supplementary Fig. 8a are superimposed the experimental pattern (plain line) and our fit (dotted line) that express the high quality of our fit.

The quality of the fit of the USAXS pattern (Supplementary Fig. 8b dotted line) confirms electron microscopy observations: the nanotubes bundles are organized into a perfect hexagonal order with a nanotube interdistance of 107Å. The intensities of the first Bragg peaks can be used to calculate a low-resolution radial electron density of the nanotubes as already performed with lipid hexagonal phases[16]. The inner radius is 28.3Å and the outer radius is 54.3 Å. The electron density variation is shown on Supplementary Fig. 7b.

Fiber Diffraction Analysis : Molecular Model

The conformation and orientation of the molecules in the nanotubes can be deduced from FTIR data, low resolution radial electron density and the $(2\pi/4.85Å^{-1})$ layer.

The analysis of the ATR-FTIR spectra demonstrates i) a turn and ii) a beta-sheet packing (Supplementary Fig. 2b). The turn has to be localized on the (D)W_6 in order to maximize the number of amides involved in beta-sheets that are intermolecular or especially intramolecular. This conformation is in agreement with (i) the (D)-residue imposing a beta-turn[17] and (ii) the radial electron density that suggests a molecular length of 26Å. No other peptide backbone conformations are in agreement with all these information.

The axial orientation of the beta-sheet layer in the fibre diffraction pattern indicates the formation of amyloid-like protofilaments that are parallel to the nanotube axis (Supplementary Fig. 8c, blue arrows). The outer excess of radial electron density indicates that W_6, Y_5, R_8 residues are on the outer part of the nanotubes so that W_3 and H_2 are in the inner part. This orientation is in agreement with the larger steric hindrance of the turn residues than the Glycine G_{10} tails.

The number of protofilaments per nanotubes is difficult to directly determine since the hexagonal lattice peaks muffle fibre diffraction of individual nanotubes. The peak observation of the equatorial layer at a resolution lower than 2Å is only possible for nanotubes exhibiting the same symmetry than the crystalline lattice at the same resolution. This is only possible with nanotubes exhibiting a 6-fold axis and, consequently, enforces that the number of protofilaments is 6n, e.g. 18, 24, 30, 36,… The protofilament interdistance could be deduced from the outer circumference = 342Å. In the literature, the interdistances observed between beta-amyloid peptidic backbones vary from 5-6Å[18] to 11-12Å[19]. These values are is in excellent agreement with 30 filaments per nanotube leading to the outer repeat distance of 11.4Å. The mean interdistance between protofilaments is 8.7Å and is fully compatible with the high intensites of the Bragg peak about $0.7Å^{-1}$. The inner repeat distance is 6.0Å. The rotation angle between filaments is 360°/30 = 12°.

The nanotubes are thus made of 30 identical parallel protofilaments. The lateral packing of these protofilaments can be identified from the fibre diffraction pattern (Supplementary Fig. 8c). Indeed, the repeat distance along the fibre axis is the size of a beta-hairpin, e.g. two beta-sheets along the H-bonds, 9.7Å. The very high quality of the fibre patterns let unambiguously see the absence of any diffuse scattering in the plane of reciprocal space corresponding to this distance. Oversaturate images offer the possibility to observe the second order $(2\pi/2.4Å^{-1})$ and third order $(2\pi/1.6Å^{-1})$ of the beta sheet (Supplementary Fig. 8c, blue arrows). The systematic extinctions of the odd layers $(2n+1).(2\pi/9.7Å^{-1})$ of the main repeat distance (9.7Å) is due to a shift of 4.85Å

between neighbouring filaments. In this case, the unit cell is not pseudo-orthorhombic but pseudo-monoclinic (Supplementary Fig. 8d). In the case of a 2D crystal, such a symmetry would affect only h+k=2n+1 Bragg peaks. But in cylindrical coordinate, the fundamental helical vector corresponds to the smallest distance along the nanotube axis[15].

On the basis, of all this information, a molecular model has been constructed (Supplementary Fig. 9) applying three rules:

- **Step 1.** A unique peptide conformation
- **Step 2.** A 9.7Å translation along the nanotube axis, e.g. the H-bonds.
- **Step 3.** A 4.85Å translation along the nanotube axis and a rotation of 12° between the protofilaments.

The convergence procedure was the following. After each step of construction i, the shortest close contacts have been identified, and the conformation for the next step (i+1) has been modified, by hand, in order to reduce the impossible close contacts. The final proposed solution does not exhibit any impossible close-contact and is in agreement with all the experimental data. The conformation energy is close to the lowest energy.

Supplementary note7: Generality of Histidine-Serine and Histidine-Aromatics conformational switches

To find our target molecules we performed a literature search with key words “pH sensitive proteins”. In parallel, we also specifically scrutinised viral fusion proteins that are known to undergo large pH-dependent conformational changes. We downloaded atomic structures for pH sensitive proteins for which both high and low pH structures are available in the Protein Data Bank. We analysed all histidines in the high pH structures as follows: We looked for 1- a serine present within 3.5-4Å with hydrogen bonding between the side chains, and 2- aromatic side chains within 3.5-4Å. For histidines that matched those criteria, we measured the corresponding side chain distances in the low pH structures (distances between histidine nitrogen and serine gamma hydroxyl and distances between the closest atoms in the side chains for serines and aromatics, respectively).

Viral fusion proteins of two unrelated classes

Fusion proteins from enveloped viruses have been widely reported to undergo major pH-dependent conformational changes[20]. Histidine protonation is believed to play a major role in the corresponding mechanisms but relevant histidines have been hard to pinpoint with few exceptions[21].

Interestingly, two unrelated families of fusion proteins (so-called “class II” and class III”) have converged towards very similar structural organizations at the viral surface. In both cases, three beta-sheet domains (in yellow, red and blue on Supplementary Fig. 10 a, b & c) make up an ectodomain connected to the C-terminal transmembrane domain (in gray, Supplementary Fig. 11a) in the viral membrane (the limit of which is indicated by the horizontal black line in Supplementary Fig. 11a, b & c). An intervening segment (in magenta on Supplementary Fig. 11a & c) and the three beta-sheet domains undergo major relocation and/or refolding in the pH-dependent, multi-step pre- to post-fusion transition[22]. Class II and class III proteins are particularly interesting cases of biological pH-dependent alternative assemblies in that they form organized or semi-organized shells at high pH and hexagonal arrays at low pH. Available structural data allow assessment of the generality of aromatic-histidine-serine switches for two very divergent class II fusion proteins: those of the flaviviruses (Supplementary Fig. 11a) and alphaviruses (Supplementary Fig. 11b); and for the single class III fusion protein for which both pre- and post-fusion ectodomain structures are available: that of the rhabdoviruses (Supplementary Fig. 11c). In all three cases, both aromatic-histidine clusters and histidine-serine H-bonds are found in key functional regions known to undergo pH-dependent changes.

Thus class II and class III viral fusion proteins at least seem to have converged towards aromatic pockets and His-Ser couples as specific pH sensors for their complex conformational and oligomerization changes.

OmpG

The first non-viral example showing the His switch described in our present study is OmpG, a monomeric pore-forming protein from *Escherichia coli* outer membranes. The OmpG channel is a 14-stranded β-barrel, with short periplasmic turns and seven extracellular loops. Crystals grown at neutral pH show the channel in the open state at 2.3Å resolution[23] (Supplementary Fig. 11d). In the 2.7Å structure of crystals grown at pH 5.6, the pore is blocked by loop 6, which folds across the channel (Supplementary Fig. 11d). The rearrangement of loop 6 appears to be triggered by a pair of histidine residues (His231 and His261). At pH7.5 the centre-to-centre distance of the imidazole rings is 4.9 Å, whereas at pH 5.6, this distance increases to 13.7Å, consistent with the protonated histidines repelling one another at acidic pH.

The re-analysis of the structures shows that at neutral pH the His231 is H-bonded to the Ser218 ((Supplementary Fig. 11d, blue, distance 2.9 Å) whereas this close vicinity is lost at low pH ((Supplementary Fig. 11d, green, distance 9.9 Å).

This, and the refolding of loop 6 into a more compact conformation, might provide the energy for the unzipping of neighbouring H-bonds. At neutral pH, the two histidines would be uncharged, allowing the H-bonds to reform and loop 6 to assume its extended conformation projecting into the extracellular space.

Ficolin-M

Ficolins are soluble oligomeric proteins with lectin-like activity, assembled from collagen fibers prolonged by fibrinogen-like recognition domains. They act as innate immune sensors by recognizing conserved molecular markers exposed on microbial surfaces and thereby triggering effector mechanisms such as enhanced phagocytosis and inflammation. In humans, L- and H-ficolins have been characterized in plasma, whereas a third species, M-ficolin, is secreted by monocytes and macrophages. The structure of M-ficolin (Supplementary Fig. 11e) has been determined at high resolution (1.75–1.8Å), together with the ligand-bound structures obtained at neutral pH (1.52Å resolution) and nonbinding conformations observed at pH 5.6 (1.70Å resolution)[24]. These structures reveal how the ligand binding site is dislocated at acidic pH. This means that the binding function of M-ficolin is subject to a pH-sensitive conformational switch. Considering that the homologous ficolin B is found in the lysosomes of activated macrophages, it is proposed that this switch could play a physiological role in such acidic compartments.

References

1 X. Zhang et al., Cryo-EM structure of the mature dengue virus at 3.5-Å resolution, *Nat. Struct. Mol. Biol.* **20**, 105–110 (2013).

2 J. E. Voss et al., Glycoprotein organization of Chikungunya virus particles revealed by X-ray crystallography, *Nature* **468**, 709–712 (2010).

3 L. Li, J. Jose, Y. Xiang, R. J. Kuhn, M. G. Rossmann, Structural changes of envelope proteins during alphavirus fusion, *Nature* **468**, 705–708 (2010).

4 S. Libersou et al., Distinct structural rearrangements of the VSV glycoprotein drive membrane fusion, *J. Cell Biol.* **191**, 199–210 (2010).

5 S. Krimm and J. Bandekar, Vibrational spectroscopy and conformation of Peptides, polypeptides and Proteins, Academic Press, 1986.

6-27 Barth, A. The infrared absorption of amino acid side chains. *Prog. Biophys. Mol. Biol.* **74**, 141-173 (2000).

7 Gobeaux, F. et al. Structural Role of Counterions Adsorbed on Self-Assembled Peptide Nanotubes. *J. Am. Chem. Soc.* **134**, 723-733 (2012).

8 Kabsch, W. XSD, *Acta Crystallogr. D* **66**, 125-132 (2010).
9 Sheldrick, G. M., A short history of SHELX, *Acta Crystallogr. A* **64**, 112-122 (2008).
10 Emsley P., Lohkamp B., Scott W., Cowtan K., Feature and development of Coot, *Acta Crystallogr. D* **66**, 486-501 (2010).
11 Murshudov, G. N., Skubák, P., Lebedev, A. A., Pannu, N. S., Steiner, R. A., Nicholls, R. A., Winn, M. D., Long, F. & Vagin, A. A. REFMAC5 for the refinement of macromolecular crystal structures, *Acta Crystallogr. D* **67**, 355-367 (2011).
12 Takeuchi, H. Raman structural markers of tryptophan and histidine side chains in proteins. *Biopolymers* **72,** 305–317 (2003).
13 Takeuchi, H. & Harada, I. Normal Coordinate Analysis of the Indole Ring. *Spectrochim. Acta Part - Mol. Biomol. Spectrosc.* **42,** 1069–1078 (1986)
14 Maruyama, T. & Takeuchi, H. Effects of Hydrogen-Bonding and Side-Chain Conformation on the Raman Bands of Tryptophan-2,4,5,6,7-D(5). *J. Raman Spectrosc.* **26,** 319–324 (1995).
15 Vainshtein, B. K. Diffraction of X-rays by chain molecules. (Elsevier, Amsterdam, London, New-York, 1966).
16 C. Bottier, J. Géan, F. Artzner, B. Desbat, M. Pézolet, A. Renault, D. Marion, V. Vié, Galactosyl headgroup interactions control the molecular packing of wheat lipids in Langmuir films and in hydrated liquid-crystalline mesophases, *BBA-Biomembranes* **1768**(6), 1526-1540 (2007).
17 Valery, C. et al. Biomimetic organization: Octapeptide self-assembly into nanotubes of viral capsid-like dimension. *Proc. Natl. Acad. Sci. U.S.A.* **100**, 10258-10262 (2003).
18 Fraser & Mac Rae, Conformation in fibrous proteins and related synthetic polypeptides, Academic Press, 1973.
19 H. Inouye, P.E. Fraser, D.A. Kirschner, Structure of beta-crystallite assemblies formed by Alzheimer beta-amyloid protein analogues: analysis by x-ray diffraction, *Biophys. J.* **64,** 502-519 (1993).
20 A. Albertini, S. Bressanelli, J. Lepault, Y. Gaudin, Structure and working of viral fusion machinery, *Curr. Top. Membr.* **68**, 49–80 (2011).
21 R. Fritz, K. Stiasny, F. X. Heinz, Identification of specific histidines as pH sensors in flavivirus membrane fusion, *J. Cell Biol.* **183**, 353–361 (2008).
22 E. Baquero et al., Intermediate conformations during viral fusion glycoprotein structural transition, *Curr. Opin. Virol.* **3**, 143–150 (2013).
23 Yildiz, O., Vinothkumar, K. R., Goswami, P. & Kuhlbrandt, W. Structure of the monomeric outer-membrane porin OmpG in the open and closed conformation. *EMBO J.* **25**, 3702-3713 (2006).
24 Garlatti, V. et al. Structural basis for innate immune sensing by M-ficolin and its control by a pH-dependent conformational switch. *J. Biol. Chem.* **282**, 35814-35820 (2007).
25 Modis, Y., Ogata, S., Clements, D. & Harrison, S. C. Structure of the dengue virus envelope protein after membrane fusion. *Nature* **427**, 313-319 (2004).
26 Roche, S., Rey, F. A., Gaudin, Y. & Bressanelli, S. Structure of the prefusion form of the vesicular stomatitis virus glycoprotein G. *Science* **315**, 843-848 (2007).
